# Microbiome Dynamics of Camel Meat During Cold Storage Revealed by 16S rRNA Gene Sequencing

**DOI:** 10.3390/biology15171462

**Published:** 2026-08-27

**Authors:** Mohammed Aladhadh

**Affiliations:** Department of Food Science and Human Nutrition, College of Agriculture and Food, Qassim University, Buraydah 51452, Saudi Arabia; aladhadh@qu.edu.sa

**Keywords:** camel meat, bacterial community, 16S rRNA gene sequencing, refrigerated storage, biodiversity

## Abstract

Camel meat is an important red meat source, but culture-independent evidence of its bacterial community during refrigerated storage remains limited. No prior study has applied 16S rRNA sequencing to camel meat under aerobic chilled storage. Samples from three butcher shops were evaluated at Day 0 and Day 7 of aerobic storage at 5 °C. Thirty-eight samples were retained for diversity, compositional, and differential abundance analyses using Breakaway, DivNet, Bayesian hierarchical modeling, permutational multivariate analysis of variance (PERMANOVA), and Microbiome Multivariable Associations with Linear Models 2. Richness and Shannon diversity decreased from Day 0 to Day 7, while the Simpson index increased, indicating higher dominance. Storage duration was associated with Bray–Curtis community variation (PERMANOVA: *R*^2^ = 0.107, *p* = 0.001), whereas anatomical cut showed no significant effect; butcher source differed in multivariate dispersion. Differential abundance analysis identified 59 storage-associated amplicon sequence variants, including *Pseudomonas* enrichment and depletion of *Kocuria*, *Rothia*, and *Carnobacterium* at Day 7. These are relative-abundance findings from 16S rRNA, not culture-based microbial counts. Storage duration was the principal driver of camel meat microbiota composition, providing a compositional baseline that may inform hygiene and storage practices aimed at limiting *Pseudomonas*-associated spoilage; microbial load and spoilage were not directly assessed.

## Simple Summary

Camel meat is an important food source in many regions, but little is known about how its bacterial makeup changes during refrigerated storage before reaching consumers. Understanding these bacteria matters because some types are linked to spoilage in chilled meat. This study aimed to investigate how storage time, meat cut, and butcher shop of origin each affect bacterial composition. Samples were collected from three retail butcher shops and tested fresh and again after seven days of cold storage. Using genetic sequencing, the bacteria present in each sample were identified without needing to grow them in a laboratory. After seven days of storage, the meat had fewer types of bacteria overall, and one particular group of bacteria, commonly linked to spoilage in chilled meat, became much more common. The type of cut, such as back, leg, or shoulder, made little difference in these changes, but the butcher shop the meat came from made a noticeable difference to how consistent the bacteria were within each group of samples. These findings show that storage time, more than the cut of meat, shapes bacterial changes in camel meat, offering useful baseline information for understanding bacterial changes in the camel meat supply chain.

## 1. Introduction

Microbial spoilage remains a major constraint on the refrigerated shelf life, quality, and marketability of fresh meat [1,2]. Refrigeration slows microbial growth, but it does not prevent the development of psychrotrophic bacteria, such as *Pseudomonas*, that can grow under low-temperature storage conditions [3,4]. The bacterial communities that emerge on meat reflect initial contamination, storage temperature, oxygen availability, packaging atmosphere, handling practices, and contact with processing or retail environments [1,3,4,5]. These factors influence not only microbial load but also the composition of the spoilage-associated community that develops during storage [6].

Aerobic refrigerated storage is particularly important because oxygen availability can favor aerobic psychrotrophic taxa [4,7]. Among these, *Pseudomonas* members are frequently reported in aerobically stored chilled meat because they grow at refrigeration temperatures and compete effectively under oxygenated conditions. However, spoilage-associated communities are not uniform across storage systems [6]. Aerobic, vacuum-packaged, and modified-atmosphere conditions can select for different bacterial groups [3,5]. Processing and retail environments can also shape initial microbial profiles, and spoilage-associated bacteria may overlap between meat products and meat-processing environments [7,8,9].

Camel meat is an important red meat in the Middle Eastern, African, and Asian regions and has gained attention for its nutritional composition. Specifically, it contains ~74% moisture, ~21% protein, ~1.51% fat, and ~0.83% ash, while in beef, moisture content is ~71.52%, protein ~20.64%, fat ~6.83%, and ash ~1.35% [10]. Similarly, the nutritional content in mutton is ~73.51% moisture, ~21.62% protein, ~4.56% fat, and ~0.84% ash. According to these statistics, camel meat has significantly higher moisture content than beef and mutton and lower fat content, while providing comparable protein content; hence, it is considered a potential alternative red meat source [11,12]. Despite this relevance, microbiological research on camel meat remains less developed than comparable work on beef and mutton [10,13]. Existing camel meat studies have mainly examined culture-based microbial indicators, sensory quality, irradiation, packaging, marination, or antimicrobial preservation strategies [14,15,16]. For example, refrigerated camel meat has been evaluated after gamma irradiation, and marinated camel meat has been studied under aerobic and vacuum-packaged storage with essential oil treatments. These studies provide useful information on microbial quality and preservation, but they do not resolve the broader bacterial community structure of fresh camel meat during refrigerated storage.

Culture-independent sequencing provides a wider view of meat-associated bacterial communities than targeted culture-based enumeration [7,17]. In beef, 16S rRNA gene amplicon sequencing has been widely used to profile bacterial community composition and to detect spoilage-associated taxa, with studies identifying *Pseudomonas*, *Brochothrix*, and lactic acid bacteria as dominant members of the aerobic refrigerated meat microbiome [8,18,19].

Previous studies on camel meat provide useful information on microbial quality and preservation but offer limited insight into community-level bacterial changes during aerobic refrigerated storage [12,13,14,15]. This gap is important because fresh camel meat may enter retail storage with microbial communities shaped by both the meat matrix and the handling environment. In particular, to the best of my knowledge, no study on the microbiota of fresh camel meat using 16S rRNA gene amplicon sequencing considers storage duration, anatomical cut, and butcher-source variability together. Anatomical cuts may differ in surface exposure, tissue composition, handling, and equipment contact, while butcher source may influence baseline microbial communities through differences in sanitation, tools, surfaces, and local handling practices [9]. Accounting for these factors can help distinguish storage-associated microbial shifts from variation introduced by tissue type or retail source.

Therefore, the present study addressed this gap by investigating changes in the bacterial community in fresh camel meat collected from three retail butcher shops and three anatomical cuts, each with three biological replicates, evaluated immediately after collection and after seven days of aerobic refrigerated storage at 5 °C. Based on prior evidence that storage time, temperature, and packaging atmosphere shape bacterial community development in chilled meat [3,4,5,7], this study hypothesized that storage duration would be associated with reduced microbial richness and diversity and a measurable shift in community composition, while anatomical cut would show a comparatively limited effect given its narrower influence on surface microenvironment. The specific objectives of this study were to: (i) characterize changes in alpha diversity (richness, Shannon diversity, and the Simpson index) between Day 0 and Day 7; (ii) determine whether storage duration, anatomical cut, and butcher source were associated with overall community composition using Bray–Curtis dissimilarities; (iii) profile the taxonomic composition of the bacterial community at the phylum and genus levels across storage duration; and (iv) identify ASV features significantly associated with storage duration, anatomical cut, or butcher source using multivariable differential abundance analysis. Together, these objectives were designed to test whether storage duration is the principal driver of microbial community change in camel meat, relative to anatomical cut and butcher source.

## 2. Materials and Methods

### 2.1. Sample Collection and Storage

A total of 27 meat samples from male camels aged 6 to 8 months were collected from three randomly selected retail butcher shops in the Qassim region of Saudi Arabia in February 2025. From each location, three cuts (back, leg, and shoulder) were obtained, with three biological replicates per cut in the initial sampling design. Samples were transported at 0–4 °C and processed aseptically within 4 h of collection. A subset of each sample was collected at Day 0 and immediately processed for DNA extraction. The remaining portions were hung in a walk-in cold room maintained at 5 °C under aerobic conditions for 7 days. Cuts were hung individually with adequate spacing to avoid surface contact and cross-contamination. Randomization was not applied, as each cut was tracked to its own butcher and replicate. Relative humidity in the cold room was not recorded. After this period, samples were aseptically resampled from the corresponding cuts, totaling 54 across the two sampling time points.

### 2.2. DNA Extraction and 16S Ribosomal RNA (16S rRNA) Sequencing

The meat samples (10 g) were aseptically collected from meat surfaces and homogenized in 90 mL of sterile peptone water using a Stomacher^®^ 400 (Seward, Worthing, UK) for 2 min to detach surface microorganisms into a microbial suspension. An aliquot of 1.8 mL of the homogenate was then transferred to a sterile 2 mL microcentrifuge tube and centrifuged at 13,000× *g* for 1 min at room temperature. The resulting pellet was used for microbial DNA extraction using the DNeasy PowerFood Microbial Kit (Qiagen, Hilden, Germany) according to the manufacturer’s instructions. The V3–V4 hypervariable region of the 16S rRNA gene was amplified using the forward primer 5′-TCGTCGGCAGCGTCAGATGTGTATAAGAGACAGCCTACGGGNGGCWGCAG-3′ and reverse primer 5′-GTCTCGTGGGCTCGGAGATGTGTATAAGAGACAGGACTACHVGGGTATCTAATCC-3′. Amplicon libraries were sequenced using paired-end sequencing (2 × 250 bp) on an Illumina MiSeq platform (San Diego, CA, USA).

### 2.3. Sequence Processing and Taxonomic Assignment

Raw sequencing reads were quality-checked using FastQC v0.12.1 and MultiQC v1.32 [20,21]. Adapter sequences were removed using Cutadapt v5.1, and downstream processing was performed in QIIME2 v2025.10 [22,23]. Amplicon sequence variants (ASVs) were inferred using the DADA2 plugin v2025.10 implemented in QIIME2, including quality filtering, denoising, paired-end read merging, and chimera removal (Table S1, Sheet 1) [24]. Taxonomic classification was performed using the consensus VSEARCH v2025.10 classifier against the SILVA 138.2 reference database with a 99% sequence identity threshold [25]. Non-bacterial sequences, including Eukaryota, Archaea, and unassigned taxa, were removed before downstream analyses. Representative ASVs were aligned, and a rooted phylogenetic tree was constructed using the QIIME2 phylogeny align-to-tree-mafft-fasttree pipeline. ASVs were filtered to retain sequences within the amplicon length range of 440–485 bp [26,27]. The initial 54 samples were subjected to quality-control thresholds. Samples with fewer than 1000 reads were excluded, and 38 (26 Day 0 and 12 Day 7) were retained for downstream analyses (Figure S1, Sheet 1 and 2, and Table S1, Sheet 2) [28]. Sequencing depth coverage of these samples was assessed using rarefaction curves, with the 1000-read inclusion threshold (Figure S1, Sheet 3). Furthermore, across all 54 samples, Fisher’s exact tests were used to test the independence of exclusion status from butcher source, day, and cut to determine whether sample exclusion was randomly distributed with respect to the experimental design [29]. Pairwise independence of butcher, day, and cut across the 38 retained samples was assessed using Fisher’s exact tests to confirm the design factors were not confounded. A sensitivity analysis using a stricter 3000-read threshold (*n* = 32) was also performed to confirm the robustness of the 1000-read threshold (File S1). The raw data have been submitted to the Sequence Read Archive (SRA) at the National Center for Biotechnology Information (NCBI) under BioProject accession number (PRJNA1495186).

### 2.4. Diversity and Statistical Analysis

#### 2.4.1. Alpha Diversity

To evaluate alpha diversity, a two-step approach was employed. In the first stage, Breakaway v4.8.4 was used to estimate microbial richness [30]. Shannon diversity and the Simpson index were estimated using DivNet v0.4.1 with no covariates [31,32]. Including covariates directly would instead return identical, cell-collapsed values for every sample sharing the same covariate combination, discarding true per-sample variation. Estimation used the most abundant ASV as the base taxon and a diagonal network, which treats taxa as independent for tractability and gives a conservative variance estimate. DivNet-estimated Shannon diversity is reported on the natural-log scale, where higher values indicate greater diversity. The DivNet Simpson index is reported in its concentration form ($\sum p_{i}^{2})$, where higher values indicate greater dominance and lower diversity. Plug-in diversity metrics undercount rare taxa under incomplete sampling saturation, which biases observed diversity downward. Breakaway and DivNet were therefore used because they estimate true richness and diversity while explicitly accounting for this incomplete sampling, rather than reporting observed counts directly. The Breakaway model has no covariate structure, while DivNet’s covariate-collapsing behavior prevents covariate effects from being tested within DivNet itself. Hence, covariate effects for both estimators were tested in a second stage, using per-sample estimates and standard errors as input to the Bayesian hierarchical and betta_random() models [30,31,33,34,35].

Storage duration and tissue cut were modeled as fixed effects, and butcher source as a random effect. This tested whether richness and diversity indices were associated with these factors. Day 0 served as the baseline, and back as the reference category. Because the random-effect grouping variable (butcher) had only three levels, it limited the precision with which the between-butcher variance component could be estimated [30,31]. This also carried a risk that the estimated between-group variance (*ssq_u*) would approach zero. Bayesian hierarchical modeling with a Student-*t* likelihood for robustness to outlying samples was therefore chosen as the primary analysis for all three alpha-diversity indices [33,34,35]. It returned a full posterior for the butcher-level variance component rather than a point estimate that can degenerate to zero. The betta_random() model was retained as a sensitivity analysis to confirm that the day and cut conclusions were not sensitive to model choice [30,31]. It used the same fixed- and random-effects structure as the Bayesian model.

Among the 38 retained samples, 11 complete Day 0 to Day 7 pairs were identified by matching replicate, cut, and butcher. For each index, Breakaway-estimated richness, DivNet-estimated Shannon diversity, and the DivNet-estimated Simpson index, a Wilcoxon signed-rank test was performed across these 11 pairs [36]. The design was partially paired. Only 11 sample sets had matched Day 0 and Day 7 portions from the same original meat sample. The remaining 16 retained samples were unpaired singletons. A pairing random effect fitted across 16 single-observation levels would not be stably estimable, so pair identity was not used as a model term. The butcher source was retained as a random effect in the primary models, as in the original design. To further evaluate robustness, sensitivity analyses were conducted using Bayesian hierarchical modeling and the betta() framework, in which butcher was treated as a fixed effect alongside storage duration and cut (File S1).

#### 2.4.2. Beta Diversity

To reduce low-frequency noise, in addition to the 1000-read threshold, ASVs with a global relative abundance below 0.005% were removed before beta-diversity analysis [37]. Based on the resulting ASVs, beta diversity was assessed using Bray–Curtis dissimilarities, and principal coordinate analysis (PCoA) was performed to visualize community structure [38,39].

Differences in microbial community composition were tested using a two-pronged approach. First, as a primary analysis, a linear mixed-effects model was fitted using the lmer function in the R package lme4 v1.1.38, which allowed butcher to be included as a random intercept in line with the study design. The first three principal coordinate axes were modeled with day and cut as fixed effects and butcher as a random intercept. Further, post hoc comparisons of the PCoA axes were performed using the R library emmeans v2.0.1, with the Kenward–Roger degrees-of-freedom approximation and the Benjamini–Hochberg false-discovery-rate correction [40,41].

Secondly, as a complementary analysis, permutational multivariate analysis of variance (PERMANOVA) with adonis2() in the R vegan package v2.7.2 [42] was performed on the Bray–Curtis distance matrix to test for overall differences in community composition. Prior to this, the homogeneity of multivariate dispersion was evaluated using betadisper(), followed by permutation testing with permutest() with 999 permutations [42,43]. Dispersion was assessed separately for storage duration, tissue cut, and butcher source. Dispersion tests evaluate within-group variability, the average distance of samples to their own group centroid. They differ conceptually from PERMANOVA, which tests differences in centroid location. A significant dispersion result indicates unequal spread rather than a compositional difference. PERMANOVA models were run with 999 permutations restricted within the butcher source to account for butcher-level grouping in the sampling design. Term-wise PERMANOVA was employed for storage duration and tissue cut, and models were fitted in both day + cut and cut + day order to assess the influence of sequential term ordering. Pairwise PERMANOVA comparisons were performed using pairwise.adonis2() for storage duration and tissue cut, with permutations restricted within butcher and Benjamini–Hochberg false-discovery-rate correction applied to account for multiple testing [41,44].

PCoA-axis mixed models and PERMANOVA address distinct questions and are not interchangeable. The mixed models test whether day and cut predict variation along specific, extracted ordination axes, a reduced representation of the data. PERMANOVA tests the complete Bray–Curtis dissimilarity matrix directly, without dimensional reduction, and therefore captures compositional variation the retained axes alone may not represent. A significant day effect isolated to one PCoA axis indicates that axis carries the storage-associated signal most clearly. It does not imply that the overall compositional difference detected by PERMANOVA is reducible to that axis. Explained variance of PERMANOVA is computed across the full multidimensional distance structure but not from any single ordination axis.

To determine whether the 11 sample pairs retained greater compositional similarity to their own origin than to unrelated samples, within-pair Bray–Curtis dissimilarities (each Day 0 sample compared with its matched Day 7 sample) were compared against between-pair dissimilarities (each Day 0 sample compared with Day 7 samples from non-matched pairs) using a one-sided Wilcoxon rank-sum test [36].

### 2.5. Differential Abundance Analysis

Differential abundance analysis was performed using Microbiome Multivariable Associations with Linear Models 2 (MaAsLin2) at the phylum, genus, and ASV levels [45]. ASV counts are compositional and zero-inflated, violating the assumptions of standard analysis of variance, so MaAsLin2 was used as the multivariable framework for testing per-taxon associations with day and cut. Specifically, the phylum- and genus-level feature tables were generated by agglomerating the ASV count table using the tax_glom() function, resulting in true phylum- and genus-level tables before analysis. Total Sum Scaling (TSS) normalization and log transformation were applied, with storage duration and tissue cut as fixed effects and butcher source as a random effect to account for butcher-level variability. Day 0 served as the baseline, and the back as the reference level. Benjamini–Hochberg false-discovery-rate correction was applied across the tested features, with FDR-adjusted *q* values < 0.05 considered significant [41]. Significant features at the true phylum, true genus, and ASV levels were retained for biological interpretation. Additionally, MaAsLin2 sensitivity analyses were performed by treating butcher as a fixed effect (File S1).

## 3. Results

### 3.1. Sample Retention and Design Validation

Of the 38 retained samples, 4091 amplicon sequence variants (ASVs) were used for downstream diversity and compositional analyses (Table S1, Sheet 3 and 4). Fisher’s exact tests showed that sample exclusion was independent of butcher (*p* = 0.22) and cut (*p* = 0.66), but was significantly associated with day (*p* < 0.0001), indicating that sample loss was concentrated at one storage time point. Among the 38 retained samples, butcher, day, and cut were pairwise independent, with day and cut (*p* = 0.42), day and butcher (*p* = 0.25), and cut and butcher (*p* = 1.00), confirming the retained design factors were not confounded.

### 3.2. Alpha Diversity Between Day 0 and Day 7

**Figure 1:**
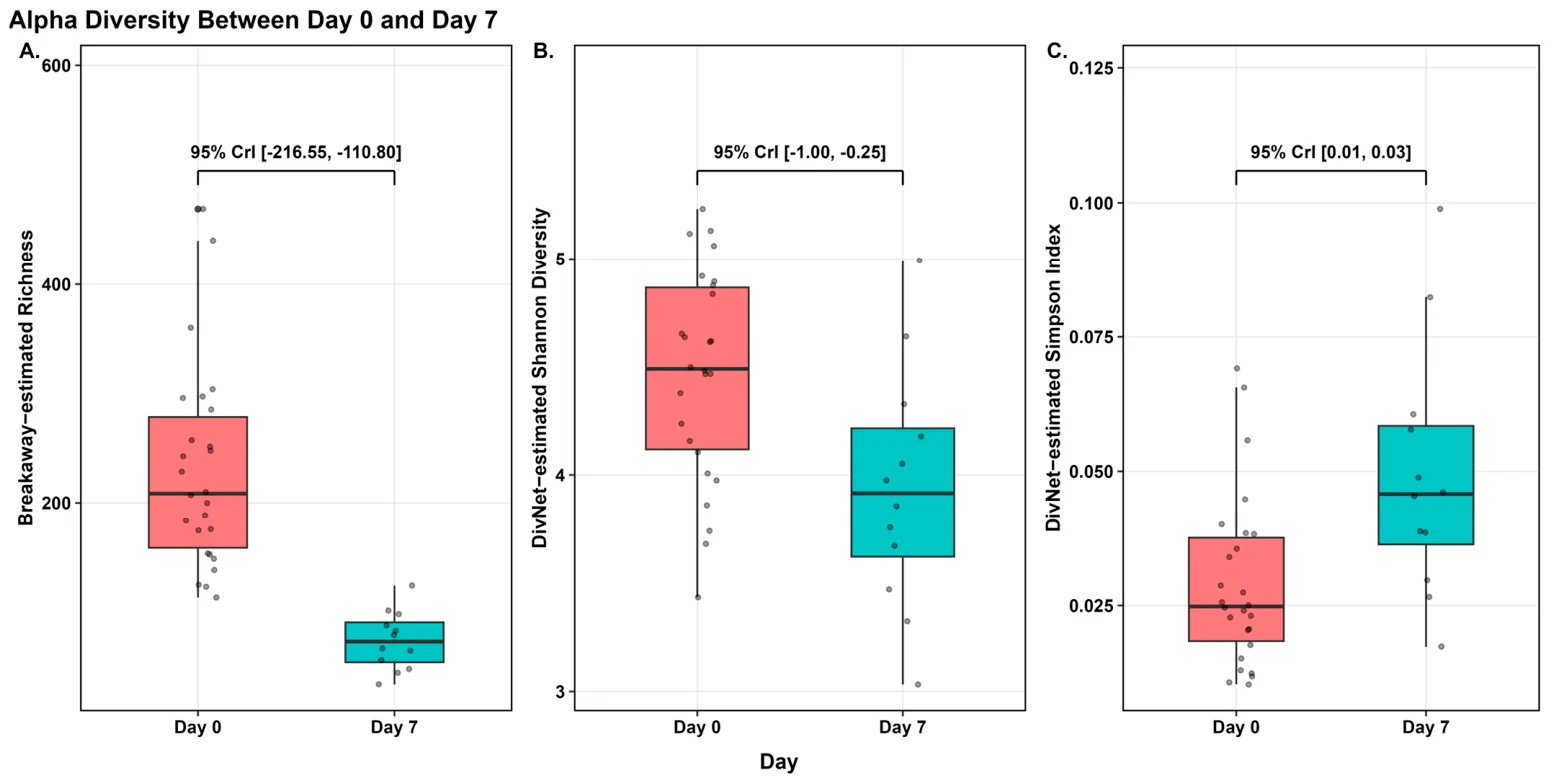
Alpha diversity between Day 0 and Day 7. (**A**) Breakaway-estimated richness, (**B**) DivNet-estimated Shannon diversity, and (**C**) DivNet-estimated Simpson index. Boxplots show the median and interquartile range of raw per-sample values pooled across butcher and cut; points are individual samples, colored by day (Day 0 salmon, Day 7 teal). Day and cut were treated as fixed effects and butcher as a random effect. The bracket reports the 95% Bayesian credible interval (CrI) for the Day 7 versus Day 0 effect, which accounts for cut and butcher and so is not a direct comparison of the two boxes.

**Figure 2:**
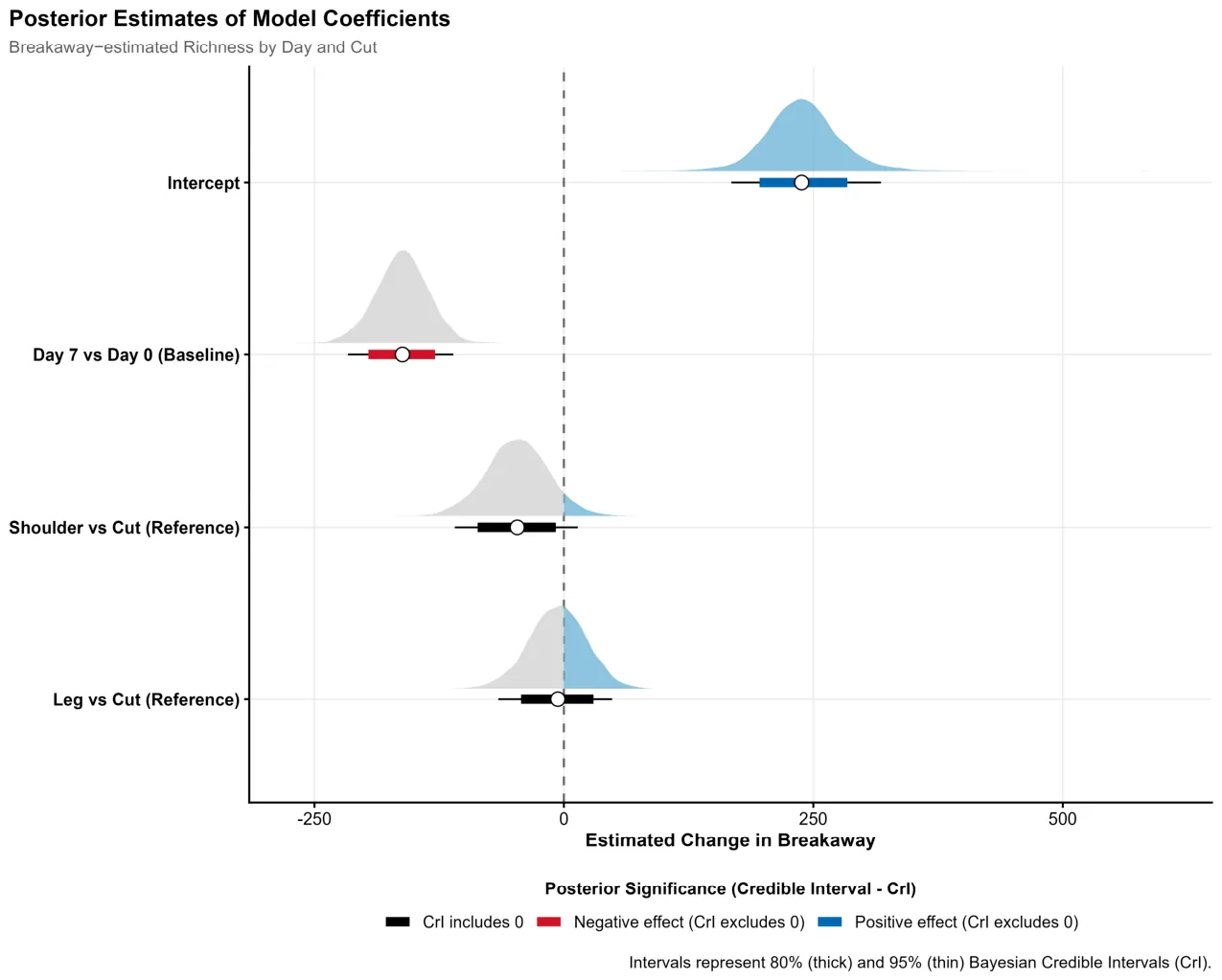
Posterior estimates of model coefficients for Breakaway-estimated richness. Bayesian hierarchical modeling with a mixed-effects model (Student-*t* likelihood) with day and cut treated as fixed effects and butcher source as a random effect. Day 0 serves as the baseline, and back is the reference category. Points represent posterior medians; thick and thin horizontal bars represent 80% and 95% Bayesian credible intervals (CrI), respectively. Blue color indicates a positive effect and red a negative effect where the 95% CrI excludes zero; black indicates the CrI includes zero.

Breakaway-estimated richness ranged from 34.17 to 469.17 across samples, with a median of 163.74 (*IQR*: 99.13 to 246.67) (Figure 1 and Table S2, Sheet 1). In a Bayesian hierarchical model with butcher as a random effect, Day 7 was associated with credibly lower richness than Day 0 (*β* = −162.20, *95% CrI* [−216.55, −110.80], *Rhat* = 1.00), whereas 95% credible intervals for meat cut effects included zero (Figure 2 and Table S2, Sheet 2). As a sensitivity analysis, a mixed-effects model with butcher as a random effect was conducted, and it was directionally consistent with the primary Bayesian model (Table S2, Sheet 3).

**Figure 3:**
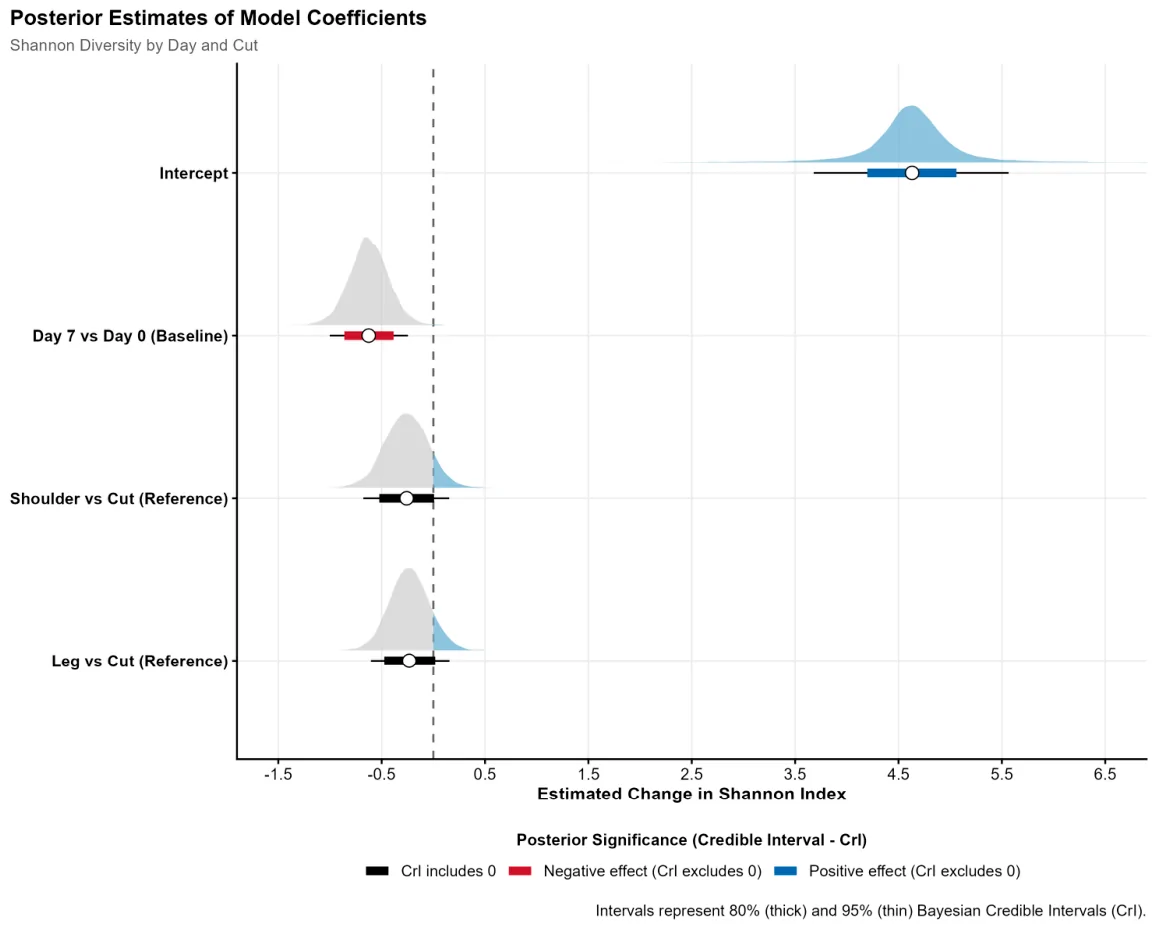
Posterior estimates of model coefficients for DivNet-estimated Shannon diversity. Coefficients from a Bayesian hierarchical model (Student-*t* likelihood) with day and cut as fixed effects and butcher as a random effect. Day 0 serves as the baseline, and back is the reference category. Points represent posterior medians; thick and thin horizontal bars represent 80% and 95% Bayesian credible intervals (CrI), respectively. Blue represents a positive effect, red a negative effect, and black indicates the CrI includes zero; the 95% CrI excludes zero.

DivNet-estimated Shannon diversity ranged from 3.03 to 5.23 across all 38 retained samples, with a median of 4.35 (*IQR*: 3.89 to 4.65) (Table S2, Sheet 4). In a Bayesian hierarchical model with butcher as a random effect, Shannon diversity was credibly lower on Day 7 than on Day 0 (*β* = −0.62, *95% CrI* [−1.00, −0.25], *Rhat* = 1.00), whereas 95% credible intervals for meat cut effects contained zero (Figure 3 and Table S2, Sheet 5). The directionality of the model was further evaluated using a sensitivity check in which a mixed-effects model with butcher as a random effect showed the same direction of effect as the primary Bayesian model (Table S2, Sheet 6).

**Figure 4:**
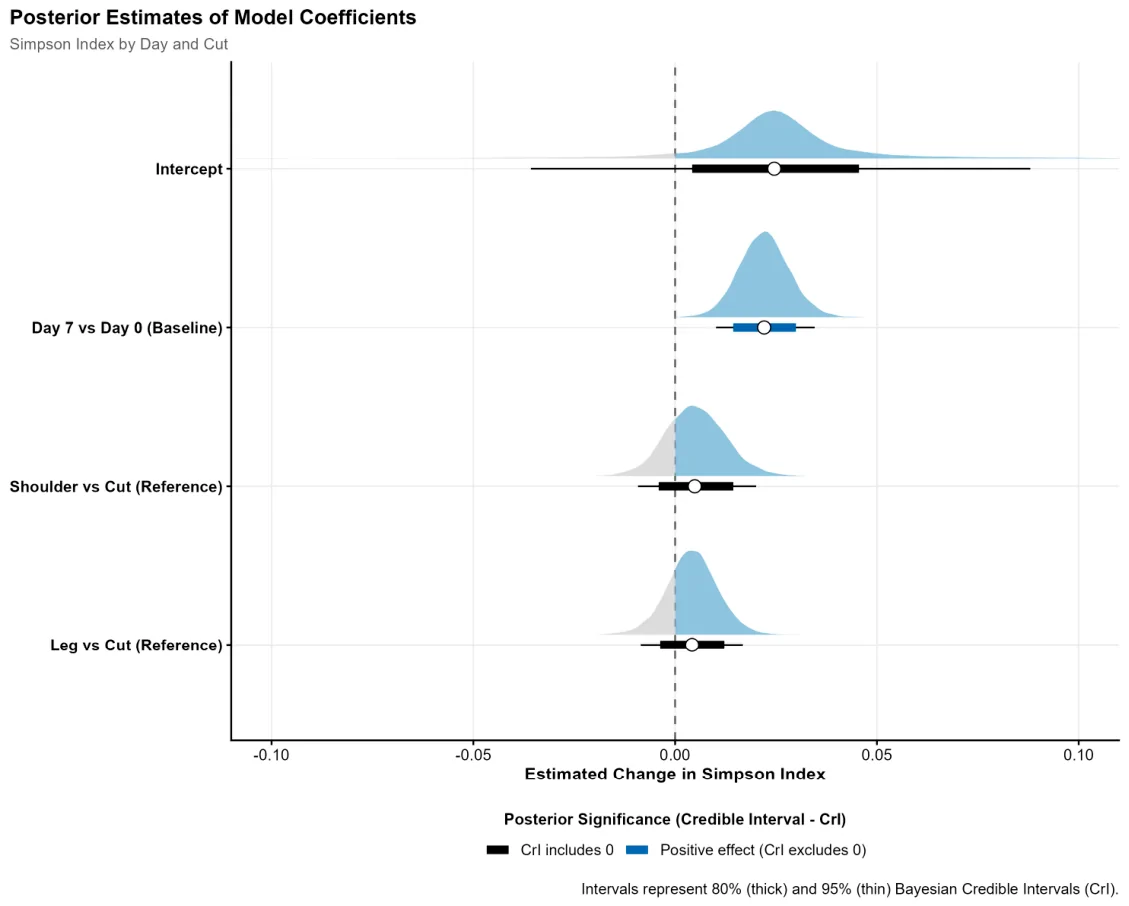
Posterior estimates of model coefficients for DivNet-estimated Simpson index. Coefficients from a Bayesian hierarchical model (Student-*t* likelihood) with day and cut as fixed effects and butcher as a random effect. Day 0 serves as the baseline, and back is the reference category. Points represent posterior medians; thick and thin horizontal bars represent 80% and 95% Bayesian credible intervals (CrI), respectively. Blue indicates a positive effect, where the 95% CrI excludes zero; black indicates the CrI includes zero.

The Simpson index estimated by DivNet ranged from 0.01 to 0.10 across all samples, with a median of 0.03 (*IQR*: 0.02 to 0.05). In a Bayesian hierarchical model with butcher as a random effect, the Simpson index was credibly higher on Day 7 than Day 0 (*β* = 0.02, *95% CrI* [0.01, 0.03], *Rhat* = 1.00), indicating greater dominance and lower diversity, whereas credible intervals for meat cut effects included zero (Figure 4 and Table S2, Sheet 7). To assess the directionality, a sensitivity analysis used a mixed-effects model with butcher as a random effect, which was consistent with the primary Bayesian model (Table S2, Sheet 8).

**Figure 5:**
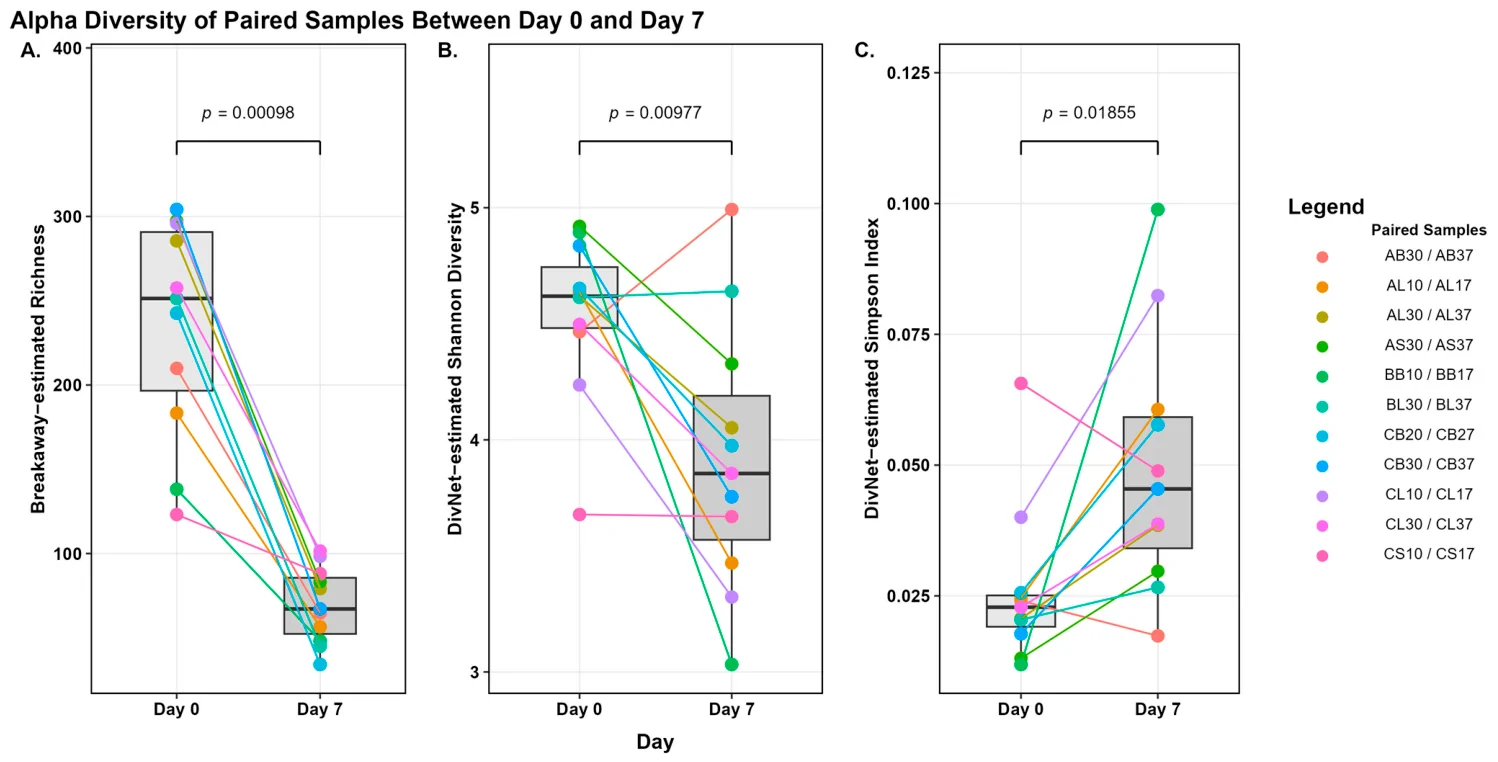
Alpha diversity of the 11 matched Day 0 to Day 7 sample pairs (22 of the 38 retained samples). (**A**) Breakaway-estimated richness, (**B**) DivNet-estimated Shannon diversity, and (**C**) DivNet-estimated Simpson index. Boxplots show the median and interquartile range at each time point; points are individual samples, and connecting lines join the two members of each pair, colored by pair as shown in the legend. *p* values are from two-sided Wilcoxon signed-rank exact tests across the 11 pairs represented.

Wilcoxon signed-rank exact tests showed significant differences across the 11 matched Day 0 to Day 7 pairs for all three alpha-diversity indices. This subset of samples is distinct from the Bayesian hierarchical and betta_random() models reported above, which use the full 38-sample retained dataset. Breakaway-estimated richness (*V* = 66, *p* = 0.00098) and DivNet-estimated Shannon diversity (*V* = 61, *p* = 0.00977) were lower on Day 7, whereas the DivNet-estimated Simpson index was higher (*V* = 7, *p* = 0.01855), which indicates greater dominance and lower diversity (Figure 5 and Table S2, Sheet 9).

### 3.3. Compositional Variation in Microbial Communities Between Day 0 and Day 7

Pairwise Bray–Curtis dissimilarities calculated across 703 sample pairs ranged from 0.192 to 1.000, with a mean of 0.808 and a median of 0.879 (Table S3, Sheet 1). Lower dissimilarities were observed among some samples from the same butcher and storage time point, whereas several Day 0 versus Day 7 comparisons reached the maximum Bray–Curtis value of 1.000, indicating compositional divergence following refrigerated storage.

As a primary analysis, beta diversity was assessed using principal coordinates analysis of Bray–Curtis dissimilarities, with the first three axes explaining 24.5%, 18.1%, and 11.0% of the variation, respectively. Each axis was modeled using a linear mixed-effects model, with day and cut as fixed effects and butcher as a random intercept. Day 7 separated significantly from Day 0 along PCoA2 (*difference* = −0.321, *SE* = 0.071, *t*(33.7) = −4.52, *p* < 0.0001), whereas PCoA1 (*p* = 0.60) and PCoA3 (*p* = 0.12) showed no significant day separation (Table S3, Sheet 2 and 3). Tissue cut was non-significant on all three axes (all pairwise comparisons *p* ≥ 0.20); therefore, storage duration separated communities only along PCoA2. Multivariate dispersion did not differ significantly by storage duration (*F* = 0.259, *p* = 0.607) or anatomical cut (*F* = 0.087, *p* = 0.910) (Figure S2, Sheet 1 and Table S3, Sheet 4). However, dispersion differed significantly among butcher sources (*F *= 8.766, *p *= 0.001), indicating unequal within-group variability across butcher groups. PERMANOVA with permutations restricted within butcher source identified storage duration as a significant contributor to microbial community variation (*R*^2^ = 0.107, *F *= 4.292, *p *= 0.001) (Table S3, Sheet 5). This effect remained significant when storage duration was fitted after anatomical cut in sequential testing (*R*^2^ = 0.101, *F *= 4.049, *p *= 0.001). Anatomical cut did not significantly affect overall community composition after accounting for storage duration. Pairwise PERMANOVA with Benjamini–Hochberg false-discovery-rate correction showed that the model remained significant between Day 0 and Day 7 microbial communities when anatomical cut was retained in the model (*R*^2^ = 0.153, *F *= 2.047, *FDR-adjusted p *= 0.001) (Table S3, Sheet 6). Because the anatomical cut was not significant in the term-wise PERMANOVA after accounting for storage duration, cut-based pairwise comparisons were interpreted as exploratory. The models were significant for back versus leg (*R*^2^ = 0.198, *F *= 2.964, *FDR-adjusted p *= 0.001) and back versus shoulder (*R*^2^ = 0.129, *F *= 1.557, *FDR-adjusted p *= 0.003), whereas the overall model for leg versus shoulder was not significant.

A one-sided Wilcoxon rank-sum test showed that matched pairs retained slightly greater compositional similarity to their own origin than to unrelated samples. Within-pair Bray–Curtis dissimilarities were significantly lower than between-pair dissimilarities (W = 396, *p* = 0.030; median 0.861 within-pair, 0.905 between-pair). The difference was small, with both medians above 0.86, indicating a weak but significant retention of pair similarity over storage (Table S3, Sheet 7).

**Figure 6:**
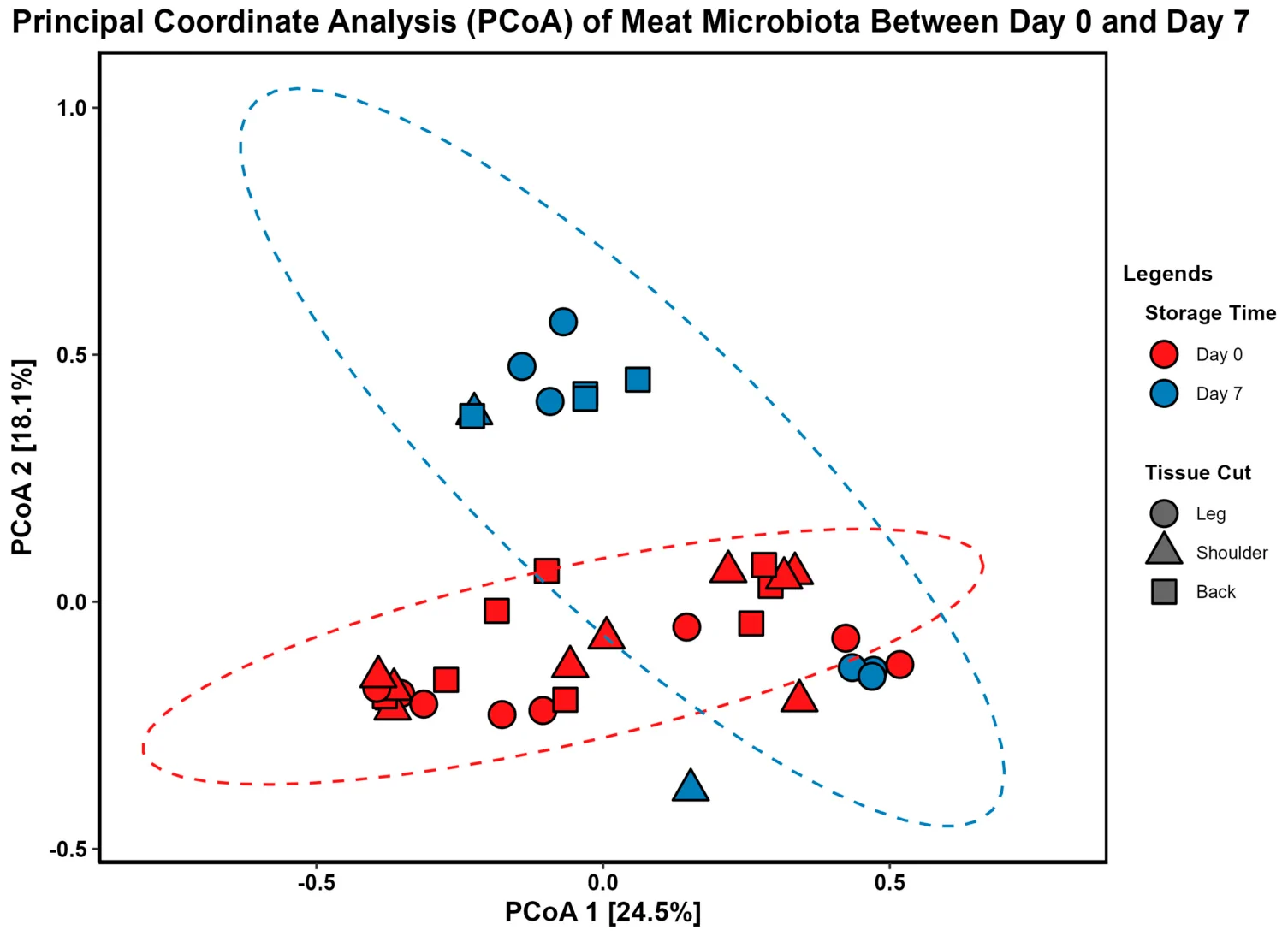
Principal coordinate analysis (PCoA) of microbial community composition based on Bray–Curtis dissimilarities. Samples are colored by storage duration and shaped according to tissue cut. PCoA1 and PCoA2 explained 24.5% and 18.1% of the total variation. PCoA1 reflected between-butcher variation rather than storage duration, whereas PCoA2 carried the storage-associated separation. Dashed ellipses represent 95% confidence regions for each storage-duration group in the two-dimensional ordination space.

Principal coordinate analysis (PCoA) based on Bray–Curtis dissimilarities showed a storage-duration-associated shift along PCoA2 (Figure 6). PCoA1 and PCoA2 explained 24.5% and 18.1% of the total variation, respectively; PCoA1 reflected between-butcher variation rather than storage duration. PERMANOVA based on the full Bray–Curtis distance matrix identified a significant storage-duration effect on microbial community composition. Butcher-stratified ordination plots are provided in Supplementary Materials (Figure S2, Sheet 2).

### 3.4. Taxonomic Composition and Relative Abundance of Dominant Microbial Taxa Across Storage Duration

**Figure 7:**
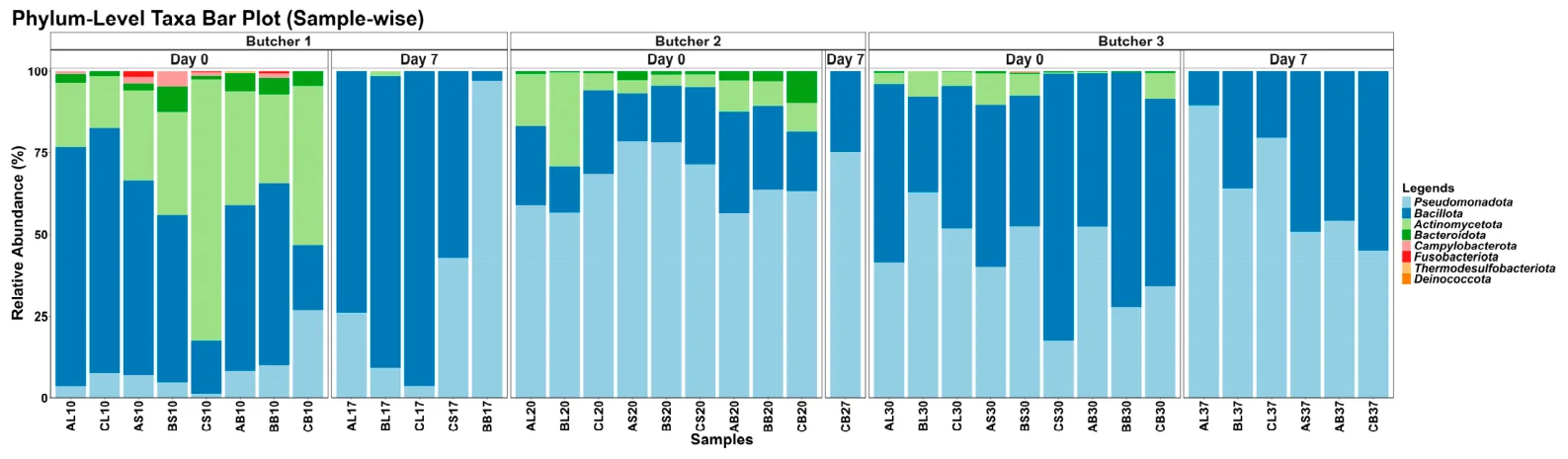
Phylum-level taxonomic composition of camel meat microbiota. Stacked bars show the percentage relative abundance of bacterial phyla per sample, faceted by butcher source (1 to 3) and storage day (Day 0, Day 7). Each bar is one sample; colors denote phyla as in the legends.

**Figure 8:**
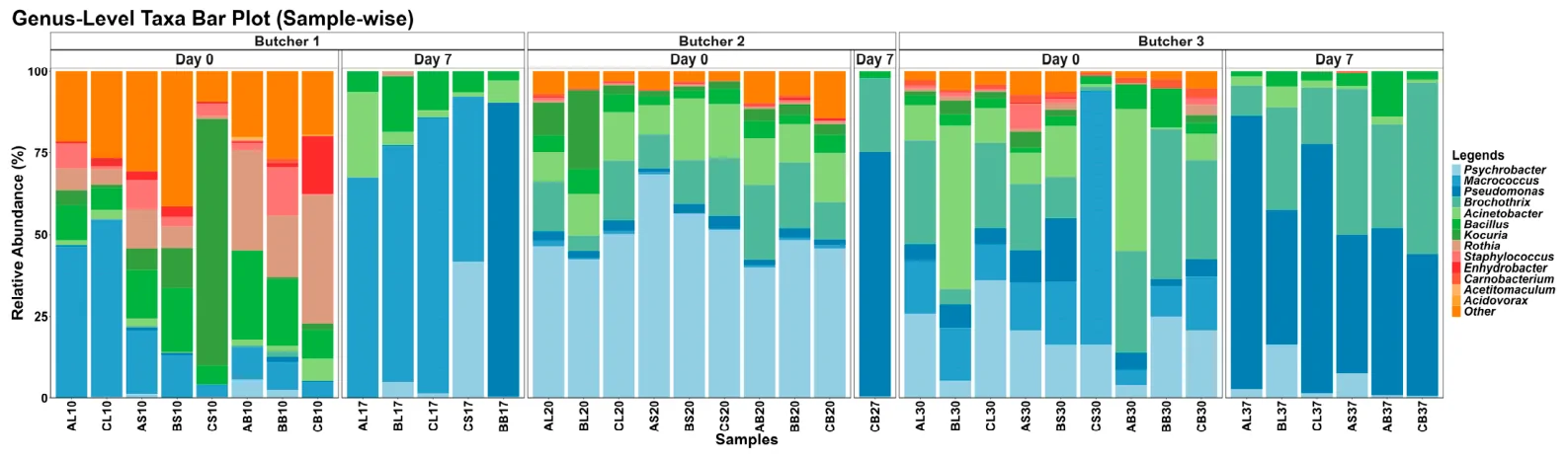
Genus-level taxonomic composition of camel meat microbiota. Stacked bars show the percentage relative abundance of bacterial genera per sample, faceted by butcher source (1 to 3) and storage day (Day 0, Day 7). Each bar is one sample; colors denote the most abundant genera as in the legends, with remaining lower-abundance genera collapsed into Other.

Taxonomic profiling showed storage-associated shifts in the relative abundance of dominant microbial taxa (Figure 7 and Figure 8). At the phylum level, communities were mainly represented by *Bacillota* and *Pseudomonadota*, with lower relative abundances of *Actinomycetota*, *Bacteroidota*, *Fusobacteriota*, *Campylobacterota*, *Deinococcota*, and *Thermodesulfobacteriota* (Table S4, Sheet 1). Day 0 samples showed more variable phylum-level profiles, with contributions from *Bacillota*, *Pseudomonadota*, and *Actinomycetota*. Following refrigerated storage, *Pseudomonadota* showed higher relative abundance in several Day 7 profiles, exceeding 70% in selected back and leg samples.

At the genus level, the selected dominant genera included *Psychrobacter*, *Macrococcus*, *Pseudomonas*, *Brochothrix*, *Acinetobacter*, *Bacillus*, *Kocuria*, *Rothia*, *Staphylococcus*, *Enhydrobacter*, *Carnobacterium*, *Acetitomaculum*, and *Acidovorax* (Table S4, Sheet 2). Day 0 communities showed higher representation of *Psychrobacter*, *Brochothrix*, *Acinetobacter*, and *Macrococcus*, whereas Day 7 profiles were characterized by higher relative abundance of *Pseudomonas* in several samples. At Day 0, *Psychrobacter* exceeded 45% relative abundance in multiple Butcher 2 samples across back, leg, and shoulder cuts. Following refrigerated storage, *Pseudomonas* increased in selected Day 7 back and leg samples, reaching up to 89.8% relative abundance. *Brochothrix* was abundant in selected samples, including one Day 7 back sample in which it reached 52.5% relative abundance. *Macrococcus* also showed high relative abundance in selected samples, exceeding 50% in Butcher 1 leg and shoulder samples.

Clustered heatmap analysis provided visual support for storage-associated differences in phylum-level composition, including higher variation in *Pseudomonadota* abundance in selected Day 7 samples (Figure S3, Sheet 1). At the genus level, the heatmap showed variation among *Pseudomonas*, *Brochothrix*, *Psychrobacter*, *Macrococcus*, and *Acinetobacter* across storage duration, tissue cut, and butcher source (Figure S3, Sheet 2).

### 3.5. Differentially Abundant ASV Features Identified by Microbiome Multivariable Associations with Linear Models 2 (MaAsLin2)

**Figure 9:**
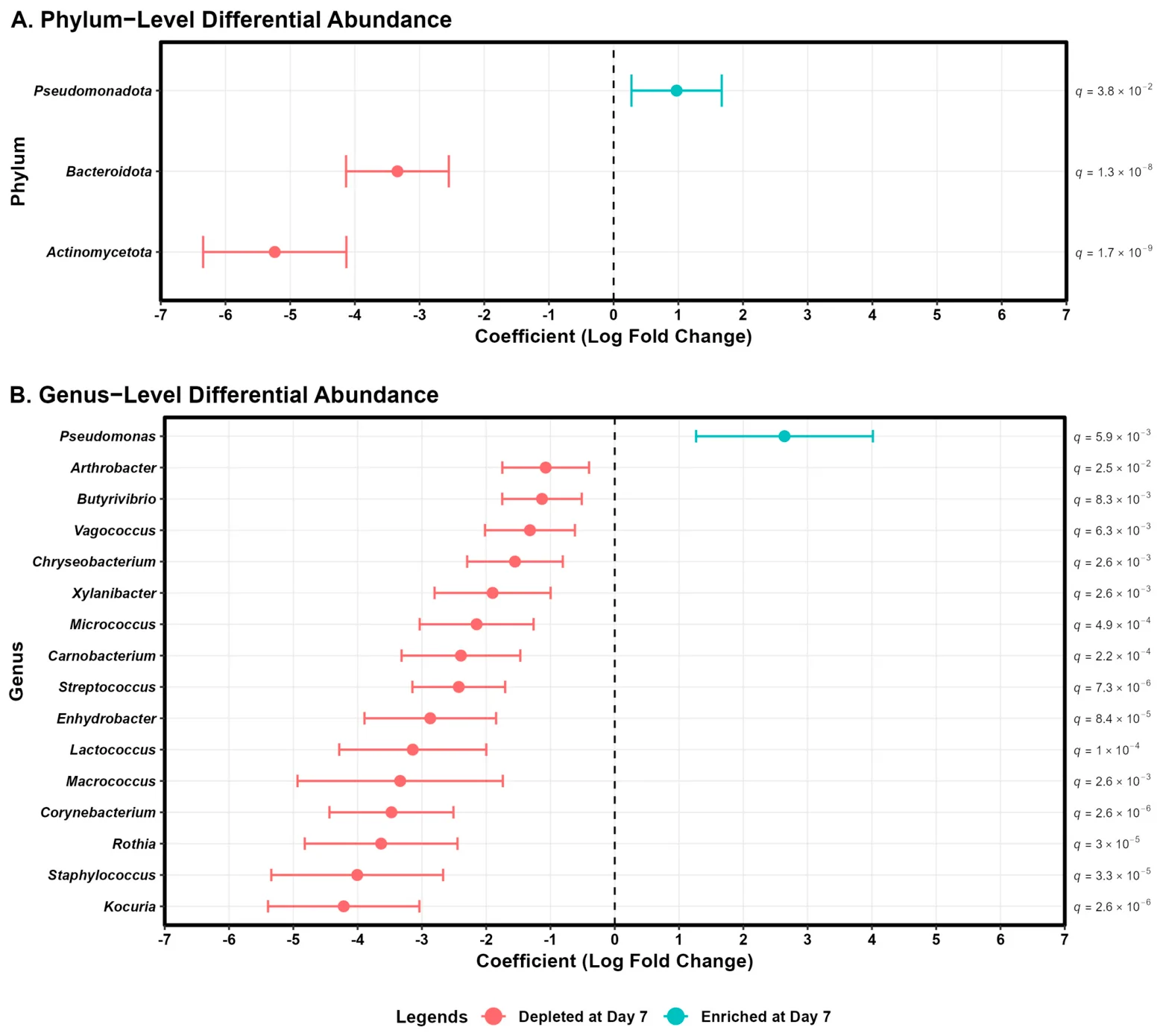
Differentially abundant bacterial taxa between Day 0 and Day 7 identified by Microbiome Multivariable Associations with Linear Models 2 (MaAsLin2). Coefficients from MaAsLin2 with day and cut as fixed effects and butcher as a random effect at (**A**) phylum and (**B**) genus level. Points are model coefficients, and horizontal bars are 95% confidence intervals calculated as the coefficient ± 1.96 × standard error, with Day 0 as the baseline. Teal marks taxa enriched at Day 7 and salmon marks taxa depleted at Day 7. Features shown passed Benjamini–Hochberg false-discovery-rate correction at *q *< 0.05, with *q* values given on the right.

Differential abundance analysis using MaAsLin2 identified storage-duration-associated changes at the true phylum, true genus, and ASV levels (Figure 9 and Figure S3, Sheet 3). At the phylum level, *Pseudomonadota* was enriched at Day 7 (*coefficient *= 0.974, *q *= 0.0378), whereas *Actinomycetota* (*coefficient *= −5.238, *q *= 1.73 × 10^−9^) and *Bacteroidota* (*coefficient *= −3.341, *q *= 1.33 × 10^−8^) were depleted (Table S4, Sheet 3). *Bacteroidota* was also lower in leg samples relative to back samples (*coefficient *= −1.197, *q *= 0.0378). At the genus level, 16 genera were significantly associated with storage duration (Table S4, Sheet 4). *Pseudomonas* was the only genus enriched at Day 7 (*coefficient *= 2.641, *q *= 0.0059). However, *Corynebacterium* (*coefficient *= −3.474, *q *= 2.63 × 10^−6^), *Kocuria* (*coefficient *= −4.216, *q *= 2.63 × 10^−6^), *Streptococcus* (*coefficient *= −2.426, *q *= 7.34 × 10^−6^), *Rothia* (*coefficient *= −3.634, *q *= 3.01 × 10^−5^), *Staphylococcus* (*coefficient *= −4.006, *q *= 3.35 × 10^−5^), *Lactococcus* (*coefficient *= −3.142, *q *= 1.00 × 10^−4^), and *Carnobacterium* (*coefficient *= −2.393, *q *= 2.16 × 10^−4^) were depleted at Day 7. *Vagococcus* was lower in shoulder samples relative to back samples (*coefficient *= −1.263, *q *= 0.0250).

At the ASV level, 59 ASVs were significantly associated with storage duration (*FDR-adjusted q *< 0.05), whereas no ASVs were significantly associated with anatomical cut (Table S4, Sheet 5). The most significant ASVs belonged to *Pseudomonadota* (45/59), followed by *Bacillota*, *Actinomycetota*, and *Bacteroidota*. Twenty-three *Pseudomonas* ASVs were enriched at Day 7, whereas ASVs assigned to *Acinetobacter*, *Psychrobacter*, *Kocuria*, *Carnobacterium*, *Macrococcus*, *Lactococcus*, and *Chryseobacterium* were depleted following storage.

## 4. Discussion

This study examined bacterial community changes in camel meat during seven days of aerobic refrigerated storage at 5 °C. Storage duration emerged as the primary factor shaping community structure, while anatomical cut showed limited evidence of a global effect. Butcher source was linked to multivariate dispersion rather than composition. These patterns place camel meat within the storage-driven microbial succession documented in other red meats, while reflecting its specific matrix properties.

Sample retention was uneven across the design, with exclusion concentrated at a single storage time point. Reduced power lowers the chance of detecting a true effect rather than inflating one, so the reported Day 7 effects likely understate the real magnitude of change. This same asymmetry restricted the paired analyses to the 11 complete Day 0–Day 7 sample pairs. The subset did not fully resolve the power loss. Pairwise independence of the design factors nonetheless confirms that the downstream models were not fitted on a confounded design.

The joint reduction in richness and Shannon diversity, alongside higher Simpson dominance, describes a community concentrated in fewer taxa by Day 7. A similar pattern appears in beefsteaks stored aerobically at 4 °C, where a highly diverse carcass community of over 600 operational taxonomic units collapsed to a few dominant taxa within seven days [46]. This parallel suggests that aerobic cold storage imposes a broadly conserved ecological filter across red meat types despite differences in matrix composition.

Storage duration drove compositional turnover, while butcher source contributed a separate and independent component of variation. The unequal dispersion among butcher sources indicates that community variability was not uniform across retail outlets, even though composition itself did not differ. This is consistent with butcher-specific handling, sanitation routines, equipment surfaces, environmental exposure, or initial contamination [7,9]. Supporting this, some strains in a beef processing study were observed in the final beef product and were also present on processing equipment and drains [47]. Farm floors, workers’ gloves, and carcass splitters have likewise been identified as major contamination sources across the beef supply chain. Such shop-level inputs would introduce heterogeneous starting communities without imposing a shared compositional signature, which matches the dispersion-only effect observed in this study. Butcher source is therefore better interpreted as a source of variability than as a direct compositional driver in camel meat.

The main taxonomic change after storage was enrichment of *Pseudomonadota* at the phylum level and *Pseudomonas* at the genus level. This agrees with previous reports describing psychrotrophic *Pseudomonas* as important members of the microbiota of aerobically stored chilled meat [3,4,9]. The same dominance occurs across meats, with high relative abundance in beef after seven days at 4 °C under aerobic conditions [5,46]. *Staphylococcus* and *Streptococcus* were identified as core microbiota in fresh meat and processing environments, with higher levels in environmental swabs than in spoiled meat [8]. *Kocuria* and *Corynebacterium* were likewise isolated from aerobic beef samples but were not dominant spoilage organisms [48]. The genera depleted here therefore trace to processing environments rather than to an established meat-associated community. *Corynebacteriaceae* was abundant in carcass swabs but was displaced as *Pseudomonas* became dominant, and *Kocuria* present after chilling was subsequently overtaken by *Pseudomonas* at one processing plant [46,48]. *Staphylococcus*, *Kocuria*, and *Streptococcus* also correlated negatively with the ASVs that became predominant during vacuum storage [49]. The depletion observed in camel meat therefore follows the same displacement of environmentally derived colonizers by psychrotrophic taxa. A comparable outcome occurred in tray-packaged mutton, which spoiled by Day 7 at 4 °C with *Pseudomonas* and *Brochothrix* considered primarily responsible [50]. Storage-associated enrichment was consistent across samples only for *Pseudomonas*, while other abundant genera varied by individual sample. This points to a shared successional endpoint reached through variable intermediate community states.

High water-holding capacity and nutrient availability are generally known drivers of microbial growth on meat surfaces [7]. This moisture-rich, low-fat matrix may similarly support the aerobic growth pattern observed for *Pseudomonas* at Day 7 in this study. *Carnobacterium*, a lactic acid bacterium with some cold tolerance, was also depleted, indicating that cold tolerance alone did not determine persistence [17,51]. This matches the established ecology of the genus, which predominates in chilled vacuum or modified-atmosphere meat but competes poorly under aerobic refrigeration. *Carnobacterium maltaromaticum*, for instance, spoils meat aerobically at 7 °C but only after prolonged vacuum storage at 2 °C [5,51]. Packaging atmosphere, rather than temperature alone, therefore determines its competitive success. Similarly, in beef stored under different packaging conditions, *Pseudomonas* dominated in air while lactic acid bacteria dominated in modified atmospheres [5]. A combination of psychrotrophy and aerobic metabolism thus gave *Pseudomonas* an advantage that lactic acid bacteria did not share [5,8,52]. Oxygen availability, rather than cold tolerance alone, appears to determine which psychrotrophic taxa dominate the camel meat surface. At broader taxonomic resolution, this succession reflects a shift from a mixed mesophilic and psychrotrophic community at Day 0 toward a psychrotroph-dominated one by Day 7 [3,4,46].

Anatomical cut showed a weaker association with microbial composition than storage duration. Pairwise cut comparisons suggested differences between some cuts, but these were interpreted as exploratory because the omnibus effect was not significant. These findings may indicate localized taxon-level differences, but they do not establish a broad cut-specific community pattern. Cut therefore appears to influence individual taxa without restructuring the community as a whole.

This study provides a controlled assessment of refrigerated aerobic storage, though several design and analytical aspects should be considered when interpreting the findings. These limitations fall into three main categories: study design and sampling, 16S rRNA sequencing data, and missing food-quality endpoints. Within study design and sampling, an a priori power calculation was not performed. This study was designed as an exploratory microbiome study to characterize shifts in the bacterial community in camel meat during refrigerated storage. Practical constraints determined the sample size: three retail butcher shops were the maximum accessible within the study region. Data collection was limited to Day 0 and Day 7, capturing the net storage effect but not the intermediate trajectory of microbial succession. The retained dataset was modest in size and unbalanced across storage duration and butcher source. This limited evaluation of interaction effects, including butcher-by-day and cut-by-day, and subtle cut-specific patterns. The findings are specific to aerobic refrigerated storage at 5 °C and should not be generalized to vacuum-packaged or modified-atmosphere-packaged meat without comparative analysis. The 16S rRNA sequencing data provide relative abundance profiles with limited species-level resolution, since short-read V3–V4 cannot separate closely related bacterial taxa reliably [53,54]. Interpretations in the present study should be limited to the ASV or genus level. Species-level claims require additional species-resolved methods, such as full-length 16S rRNA sequencing, shotgun metagenomics, targeted isolate sequencing, or validated species-specific assays [55,56,57,58]. Relative-abundance data also mean that the observed decreases cannot be read as direct evidence of bacterial death or elimination. Although homogenates were processed without incubation, sequencing does not separate DNA from viable, dormant, or dead cells. These reductions therefore indicate community turnover rather than confirmed population loss. Absolute-abundance methods, such as qPCR, culture-based enumeration, or internal-standard-based sequencing, would be needed to distinguish population decline from compositional replacement [15]. Coverage was restricted to bacterial composition, so Internal Transcribed Spacer (ITS) or 18S rRNA sequencing would provide additional insight into fungal members of the camel meat microbiome. Regarding missing food-quality endpoints, sensory evaluation, volatile organic compounds (VOCs), pH, total viable counts, and specific spoilage-organism counts were not measured. The reported community changes therefore represent storage-related microbiome shifts rather than direct evidence of off-odors, slime formation, spoilage phenotype, or shelf-life endpoints.

Future work should include prospective statistical power calculations when warranted to further validate these findings. Studies should also extend the storage time course beyond Day 7 to resolve intermediate stages of microbial succession. This would identify when storage-associated taxa become established. Combining microbiome profiling with total viable counts, targeted enumeration of spoilage organisms, and qPCR-based absolute quantification would strengthen inference. Adding pH, water activity, sensory evaluation, and volatile compound analysis would link compositional shifts to measurable quality changes. Comparative studies across aerobic, vacuum, and modified-atmosphere packaging are also needed to determine how oxygen availability and packaging conditions influence community trajectories. Larger and more balanced sampling across butcher sources and tissue cuts would further improve the assessment of facility-level variability and cut-specific effects.

## 5. Conclusions

Seven days of aerobic refrigerated storage at 5 °C reduced bacterial richness and Shannon diversity, while Simpson dominance increased. This indicates loss of richness and evenness alongside a community increasingly concentrated in fewer taxa. This compositional turnover was significant by PERMANOVA and isolated to a single ordination axis, distinguishing genuine storage-associated change from butcher-source variability. Phylum *Pseudomonadota* and its genus, *Pseudomonas*, were the only taxa enriched at Day 7, while several skin- and environment-associated phyla and genera were depleted. Anatomical cut showed no significant overall effect on community composition. Together, these findings establish storage duration as the principal driver of camel meat microbiota, and provide the first compositional baseline for this previously uncharacterized meat source. These are relative-abundance findings from 16S rRNA gene amplicon sequencing, describing compositional shifts rather than absolute changes in bacterial load. They do not indicate bacterial death or population decline. Future studies should combine microbial community profiling with viable counts, qPCR-based quantification, and direct spoilage or quality endpoints. This would link these compositional shifts to practical meat quality and shelf-life outcomes.

## Data Availability

The raw data supporting the conclusions of this article have been deposited in the Sequence Read Archive (SRA) at the National Center for Biotechnology Information (NCBI) with accession number PRJNA1495186, which will be publicly accessible from 1 January 2028. Meanwhile, this data will be available from the author upon request.

## Supplementary Materials

The following supporting information can be downloaded at https://www.mdpi.com/article/10.3390/biology15171462/s1; all provided materials are in spreadsheet form to manage properly (Figures S1–S4 and Tables S1–S5): Figure S1, Sheet 1: Read-depth distribution across all 54 samples and sample retention following sequencing quality control; Figure S1, Sheet 2: Sample retention flow diagram; Figure S1, Sheet 3: Rarefaction curves of microbial communities across retained samples; Figure S2, Sheet 1: Homogeneity of multivariate dispersion based on Bray–Curtis dissimilarities; Figure S2, Sheet 2: Principal coordinate analysis of microbial community composition stratified by butcher source; Figure S3, Sheet 1: Phylum-level clustered heatmap of microbial community composition across butcher sources and sampling days; Figure S3, Sheet 2: Genus-level clustered heatmap of microbial community composition across butcher sources and sampling days; Figure S3, Sheet 3: Amplicon sequence variants-level differential abundance between day 0 and day 7; Figure S4, Sheet 1: Posterior estimates of model coefficients for Breakaway-estimated species richness; Figure S4, Sheet 2: Posterior estimates of model coefficients for Shannon diversity; Figure S4, Sheet 3: Posterior estimates of model coefficients for Simpson diversity; Figure S4, Sheet 4: Phylum-level phylogenetic relationships among bacterial taxa detected in the study; Figure S4, Sheet 5: Genus-level phylogenetic relationships among bacterial taxa detected in the study; Table S1, Sheet 1: Sequencing quality-control metrics and sample retention status for all 54 samples; Table S1, Sheet 2: Distribution of the 38 retained samples by butcher source, sampling day, and meat cut; Table S1, Sheet 3: Amplicon sequence variant count matrix for the 38 retained samples; Table S1, Sheet 4: Representative nucleotide sequences of amplicon sequence variants identified in the retained samples; Table S2, Sheet 1: Breakaway estimates of species richness for individual samples; Table S2, Sheet 2: Betta random-effects model of Breakaway-estimated species richness by sampling day and meat cut; Table S2, Sheet 3: Bayesian hierarchical mixed-effects model of Breakaway-estimated species richness by sampling day and meat cut; Table S2, Sheet 4: Sample-level Shannon and Simpson diversities estimated by using DivNet; Table S2, Sheet 5: Betta random-effects model of Shannon diversity by sampling day and meat cut; Table S2, Sheet 6: Bayesian hierarchical mixed-effects model of Shannon diversity by sampling day and meat cut; Table S2, Sheet 7: Betta random-effects model of Simpson index by sampling day and meat cut; Table S2, Sheet 8: Bayesian hierarchical mixed-effects model of Simpson diversity by sampling day and meat cut; Table S2, Sheet 9: Paired comparisons of alpha-diversity measures between day 0 and day 7; Table S3, Sheet 1: Pairwise Bray–Curtis dissimilarity matrix for the retained samples; Table S3, Sheet 2: Linear mixed-effects models of PCoA axes derived from Bray–Curtis dissimilarities; Table S3, Sheet 3: Estimated marginal means and pairwise post hoc comparisons of PCoA axes by sampling day and meat cut; Table S3, Sheet 4: Homogeneity of multivariate dispersion by sampling day, meat cut, and butcher source; Table S3, Sheet 5: Permutational multivariate analysis of variance (PERMANOVA) of microbial community composition by sampling day and meat cut; Table S3, Sheet 6: Pairwise PERMANOVA comparisons by sampling day and meat cut; Table S3, Sheet 7: Comparison of within-pair and between-pair Bray–Curtis dissimilarities; Table S4, Sheet 1: Phylum-level taxonomic composition and relative abundance across retained samples; Table S4, Sheet 2: Genus-level taxonomic composition and relative abundance across retained samples; Table S4, Sheet 3: Phylum-level differential abundance analysis using MaAsLin2 with butcher source modeled as a random effect; Table S4, Sheet 4: Genus-level differential abundance analysis using MaAsLin2 with butcher source modeled as a random effect; Table S4, Sheet 5: ASV-level differential abundance analysis using MaAsLin2 with butcher source modeled as a random effect; Table S5, Sheet 1: Breakaway estimates of species richness after application of a 3,000-read threshold; Table S5, Sheet 2: Betta random-effects model of Breakaway-estimated species richness after application of the 3,000-read threshold; Table S5, Sheet 3: Homogeneity of multivariate dispersion after application of the 3,000-read threshold; Table S5, Sheet 4: PERMANOVA of microbial community composition after application of the 3,000-read threshold; Table S5, Sheet 5: Betta fixed-effects model of Breakaway-estimated species richness by sampling day, meat cut, and butcher source; Table S5, Sheet 6: Bayesian fixed-effects model of Breakaway-estimated species richness by sampling day, meat cut, and butcher source; Table S5, Sheet 7: Betta fixed-effects model of Shannon diversity by sampling day, meat cut, and butcher source; Table S5, Sheet 8: Bayesian fixed-effects model of Shannon diversity by sampling day, meat cut, and butcher source; Table S5, Sheet 9: Betta fixed-effects model of Simpson diversity by sampling day, meat cut, and butcher source; Table S5, Sheet 10: Bayesian fixed-effects model of Simpson diversity by sampling day, meat cut, and butcher source; Table S5, Sheet 11: Phylum-level differential abundance analysis with butcher source modeled as a random effect; Table S5, Sheet 12: Phylum-level differential abundance analysis with butcher source modeled as a fixed effect; Table S5, Sheet 13: Genus-level differential abundance analysis with butcher source modeled as a random effect; Table S5, Sheet 14: Genus-level differential abundance analysis with butcher source modeled as a fixed effect; Table S5, Sheet 15: ASV-level differential abundance analysis with butcher source modeled as a random effect; Table S5, Sheet 16: ASV-level differential abundance analysis with butcher source modeled as a fixed effect File S1: Document file contains descriptive information regarding sensitivity and additional analyses.
