# Supplementary material for: Microbiome Dynamics of Camel Meat During Cold Storage Revealed by 16S rRNA Gene Sequencing
Source: Biology (Basel). 2026 Aug 27;15(17):1462. doi: 10.3390/biology15171462 (PMC13564619; doi:10.3390/biology15171462)
Supplement: Supplementary file 1 [file biology-15-01462-s001.zip › Captions_For_Supplementary_Material_v24.pdf]

**Figure S1, Sheet 1. Read-depth distribution across all 54 samples and sample retention following sequencing quality control.**

Distribution of sequencing read counts across all 54 samples after applying sequence-length filtering and removal of reads assigned to uncategorized genera. The red dashed line marks the minimum read-depth threshold of 1,000 reads used for sample inclusion. Samples below this threshold were excluded (red) and above the threshold were retained (green).

**Figure S1, Sheet 2. Sample retention flow diagram.**

Flow diagram summarizing sample inclusion and exclusion after applying the 1,000-read quality-control threshold. Initially, 54 samples were collected and sequenced. Sixteen were excluded for fewer than 1,000 final reads (1 Day 0, 15 Day 7), and 38 were retained (26 Day 0, 12 Day 7).

**Figure S1, Sheet 3. Rarefaction curves of microbial communities across retained samples.**

Rarefaction curves showing accumulation of observed amplicon sequence variants (ASVs) with sequencing depth for each retained sample, colored by butcher source. The red dashed line marks the 1,000-read retention threshold.

**Figure S2, Sheet 1. Homogeneity of multivariate dispersion based on Bray–Curtis dissimilarities.**

Assessment of multivariate dispersion based on Bray–Curtis distances from individual samples to their corresponding group centroids according to **(A)** sampling day, **(B)** meat cut, and **(C)** butcher source. Boxplots summarize the distributions of distances within each group, with individual observations overlaid. Dispersion was tested using permutation-based tests with 999 permutations.

**Figure S2, Sheet 2. Principal coordinate analysis of microbial community composition stratified by butcher source.**

Principal coordinate analysis (PCoA) based on Bray–Curtis dissimilarities showing variation in microbial community composition between day 0 and day 7 separately for Butchers 1, 2, and 3. Colors distinguish sampling days, while point shapes denote meat cuts (leg, shoulder, or back). PCoA1 and PCoA2 explain 24.5% and 18.1% of the total compositional variation, respectively. Dashed ellipses represent 95% confidence regions for each storage-duration group in the two-dimensional ordination space.

**Figure S3, Sheet 1. Phylum-level clustered heatmap of microbial community composition across butcher sources and sampling days.**

Heatmap showing the relative abundance of bacterial phyla across retained samples arranged by butcher source and sampling day. Samples are organized into butcher-day groups, as indicated by the annotation bar above the heatmap, while phyla are hierarchically clustered according to similarities in their relative abundance profiles across samples. The logarithmic color scale ranges from absent taxa (dark blue) to progressively higher relative abundances, with reference levels of 0.01%, 0.1%, 1%, 10%, and greater than 10%.

**Figure S3, Sheet 2. Genus-level clustered heatmap of microbial community composition across butcher sources and sampling days.**

Heatmap showing genus-level relative abundance profiles across retained samples arranged by butcher source and sampling day. The annotation bar identifies the corresponding butcher-day group for each sample, while genera are hierarchically clustered according to similarities in their abundance patterns across samples. The logarithmic color scale ranges from absent taxa (dark blue) to progressively higher relative abundances, with reference levels of 0.01%, 0.1%, 1%, 10%, and greater than 10%.

**Figure S3, Sheet 3. Amplicon sequence variants-level differential abundance between day 0 and day 7.**

Differential abundance analysis of amplicon sequence variants (ASVs) was performed using MaAsLin2, with sampling day and meat cut included as fixed effects and butcher source modeled as a random effect. Day 0 serves as the baseline for sampling day. Points are model coefficients, and horizontal bars are 95% confidence intervals calculated as the coefficient ± 1.96 × standard error. Teal marks taxa enriched at day 7, and salmon marks taxa depleted at day 7. Features shown passed Benjamini–Hochberg false-discovery-rate correction at $q < 0.05$, with $q$ values given at right.

**Figure S4, Sheet 1. Posterior estimates of model coefficients for Breakaway-estimated species richness.**

Posterior estimates from a Bayesian hierarchical model evaluating Breakaway-estimated species richness with sampling day, meat cut, and butcher source included as fixed effects. Uncertainty in the sample-level richness estimates was incorporated using a Student-*t* likelihood. Points represent posterior medians, while thick and thin horizontal bars represent the 80% and 95% Bayesian credible intervals (CrIs), respectively. Day 0 serves as the baseline, while Back and Butcher 1 are the reference categories for sampling day, meat cut, and butcher source, respectively. Blue color indicates a positive effect and red a negative effect where the 95% CrI excludes zero; black indicates the CrI includes zero.

**Figure S4, Sheet 2. Posterior estimates of model coefficients for Shannon diversity.**

Posterior estimates from a Bayesian hierarchical model evaluating Shannon diversity species richness with sampling day, meat cut, and butcher source included as fixed effects. Uncertainty in the sample-level richness estimates was incorporated using a Student-*t* likelihood. Points represent posterior medians, while thick and thin horizontal bars represent the 80% and 95% Bayesian credible intervals (CrIs), respectively. Day 0 serves as the baseline, while Back and Butcher 1 are the reference categories for sampling day, meat cut, and butcher source, respectively. Blue color indicates a positive effect and red a negative effect where the 95% CrI excludes zero; black indicates the CrI includes zero.

**Figure S4, Sheet 3. Posterior estimates of model coefficients for Simpson diversity.**

Posterior estimates from a Bayesian hierarchical model evaluating Simpson diversity species richness with sampling day, meat cut, and butcher source included as fixed effects. Uncertainty

in the sample-level richness estimates was incorporated using a Student-*t* likelihood. Points represent posterior medians, while thick and thin horizontal bars represent the 80% and 95% Bayesian credible intervals (CrIs), respectively. Day 0 serves as the baseline, while Back and Butcher 1 are the reference categories for sampling day, meat cut, and butcher source, respectively. Blue color indicates a positive effect where the 95% CrI excludes zero; black indicates the CrI includes zero.

**Figure S4, Sheet 4. Phylum-level phylogenetic relationships among bacterial taxa detected in the study.**

Phylogenetic tree showing the evolutionary relationships among bacterial phyla detected in the study samples. Terminal labels identify individual phyla, while branch lengths represent evolutionary distance, expressed as substitutions per site. The tree provides general taxonomic context.

**Figure S4, Sheet 5. Genus-level phylogenetic relationships among bacterial taxa detected in the study.**

Phylogenetic tree showing the evolutionary relationships among bacterial genera detected in the study samples. Terminal labels identify individual genera, while branch lengths represent evolutionary distance, expressed as substitutions per site. The tree provides general taxonomic context.

**Table S1, Sheet 1. Sequencing quality-control metrics and sample retention status for all 54 samples.**

Sequencing and quality-control statistics for all 54 samples across successive sequence-processing stages, including raw reads, reads retained after adapter removal and quality filtering, denoised reads, merged reads, non-chimeric reads, and final read counts. Final read counts were obtained after sequence-length filtering and removal of reads assigned to uncategorized genera. Samples containing fewer than 1,000 final reads were excluded from downstream analyses, and the retention status of each sample is reported. Sample identifiers (SampleID) are encoded as position-wise, Replicate (A = Replicate A, B = Replicate B, C = Replicate C), Cut (B = Back, L = Leg, S = Shoulder), Butcher (1 = Butcher 1, 2 = Butcher 2, 3 = Butcher 3), and day (0 = Day 0, 7 = Day 7), e.g., AB10 indicates replicate A, back cut, butcher 1, day 0

**Table S1, Sheet 2. Distribution of the 38 retained samples by butcher source, sampling day, and meat cut.**

Distribution of the 38 samples retained after applying a 1,000-read quality-control threshold. Sample numbers are summarized by butcher source, sampling day (day 0 or day 7), and anatomical meat cut (back, leg, or shoulder), together with the corresponding group totals.

**Table S1, Sheet 3. Amplicon sequence variant count matrix for the 38 retained samples.**

Raw count matrix for the 38 samples retained after sequencing quality control. Rows represent individual amplicon sequence variants (ASVs), columns represent individual samples, and

each cell contains the number of sequencing reads assigned to the corresponding ASV in that sample.

**Table S1, Sheet 4. Representative nucleotide sequences of amplicon sequence variants identified in the retained samples.**

Representative nucleotide sequences of the amplicon sequence variants identified across the 38 retained samples, together with their corresponding ASV identifiers and sequence lengths.

**Table S2, Sheet 1. Breakaway estimates of species richness for individual samples.**

Sample-level species richness estimates were calculated by using Breakaway. The table reports estimated richness, the associated estimation error, lower and upper confidence bounds, and the statistical model selected by Breakaway (Kemp, Negative Binomial, and Poisson).

**Table S2, Sheet 2. Betta random-effects model of Breakaway-estimated species richness by sampling day and meat cut.**

Betta random-effects model of Breakaway-estimated richness by sampling day and meat cut as fixed effects, and butcher source as random effect. Day 0 and back serve as the reference categories.

**Table S2, Sheet 3. Bayesian hierarchical mixed-effects model of Breakaway-estimated species richness by sampling day and meat cut.**

Posterior estimates from a Bayesian hierarchical mixed-effects model evaluating Breakaway-estimated species richness according to sampling day and meat cut as fixed effects, whereas butcher source was modeled as a random effect. Uncertainty in the sample-level richness estimates was incorporated using a Student-*t* likelihood. Day 0 serves as the baseline, and Back is treated as the reference category. The table reports posterior coefficient estimates, 95% credible intervals, the among-butcher random-effect standard deviation, residual distribution parameters, Rhat, and convergence diagnostics.

**Table S2, Sheet 4. Sample-level Shannon and Simpson diversities estimated by using DivNet.**

Sample-level Shannon and Simpson diversity indices computed using DivNet. For each sample, the table reports the estimated diversity values, associated estimation errors, and lower and upper confidence bounds. All samples used the same model, “Aitchison”, to calculate Shannon and Simpson diversity indices.

**Table S2, Sheet 5. Betta random-effects model of Shannon diversity by sampling day and meat cut.**

Betta random-effects model of Shannon diversity by sampling day and meat cut as fixed effects, and butcher source as random effect. Day 0 and Back serve as the reference categories.

**Table S2, Sheet 6. Bayesian hierarchical mixed-effects model of Shannon diversity by sampling day and meat cut.**

Posterior estimates from a Bayesian hierarchical model assessing Shannon diversity by sampling day and meat cut as fixed effects, whereas butcher source was modeled as a random effect. Uncertainty in the sample-level Shannon diversity estimates was incorporated using a Student-*t* likelihood. Day 0 serves as the baseline, and Back is treated as the reference category. The table reports posterior coefficient estimates, 95% Bayesian credible intervals (CrIs), the among-butcher random-effect standard deviation, residual distribution parameters, Rhat, and convergence diagnostics.

**Table S2, Sheet 7. Betta random-effects model of Simpson index by sampling day and meat cut.**

Betta random-effects model of Simpson index by sampling day and meat cut as fixed effects, and butcher source as random effect. Day 0 and Back serve as the reference categories.

**Table S2, Sheet 8. Bayesian hierarchical mixed-effects model of Simpson diversity by sampling day and meat cut.**

Posterior estimates from a Bayesian hierarchical model assessing Simpson index by sampling day and meat cut as fixed effects, whereas butcher source was modeled as a random effect. Uncertainty in the sample-level Simpson diversity estimates was incorporated using a Student-*t* likelihood. Day 0 serves as the baseline, and Back is treated as the reference category. The table reports posterior coefficient estimates, 95% Bayesian credible intervals (CrIs), the among-butcher random-effect standard deviation, residual distribution parameters, Rhat, and convergence diagnostics.

**Table S2, Sheet 9. Paired comparisons of alpha-diversity measures between day 0 and day 7.**

Exact Wilcoxon signed-rank tests were applied on 11 complete pairs of day 0 and day 7, identified by matching Replicate, Cut, and Butcher, for Breakaway-estimated richness, Shannon diversity, and Simpson index. Separate paired tests were performed for each alpha diversity measure. The table reports the Wilcoxon signed-rank statistic and exact *p*-value for each comparison.

**Table S3, Sheet 1. Pairwise Bray–Curtis dissimilarity matrix for the retained samples.**

Pairwise Bray–Curtis dissimilarities calculated from the microbial community profiles of the 38 retained samples. Each cell represents the compositional dissimilarity between two samples, with values approaching 0 indicating increasingly similar community composition, whereas values reaching 1 represent increasingly dissimilar communities.

**Table S3, Sheet 2. Linear mixed-effects models of PCoA axes derived from Bray–Curtis dissimilarities.**

Linear mixed-effects models evaluating associations of sampling day and meat cut with PCoA1, PCoA2, and PCoA3 derived from Bray–Curtis dissimilarities. Sampling day and meat cut were modeled as fixed effects, whereas butcher source was included as a random intercept.

For each PCoA axis, the table reports the fixed-effect estimates, random-effect variance components, and associated model statistics.

**Table S3, Sheet 3. Estimated marginal means and pairwise post hoc comparisons of PCoA axes by sampling day and meat cut.**

Estimated marginal means and pairwise post hoc comparisons derived from the linear mixed-effects models of the PCoA axes. Sampling day and meat cut were included as fixed effects, while butcher source was retained as a random intercept in the underlying models. The table reports estimated marginal means, standard errors, degrees of freedom, 95% confidence intervals, pairwise contrasts, test statistics, and corresponding *p*-values, with false discovery rate adjustment applied to multiple comparisons where specified.

**Table S3, Sheet 4. Homogeneity of multivariate dispersion by sampling day, meat cut, and butcher source.**

Permutation-based tests for homogeneity of multivariate dispersion based on Bray–Curtis distances to group centroids. Separate analyses evaluated within-group dispersion by sampling day, meat cut, and butcher source, using 999 unrestricted permutations. The table reports degrees of freedom, sums of squares, mean squares, $F$ statistics, and permutation-based *p*-values.

**Table S3, Sheet 5. Permutational multivariate analysis of variance (PERMANOVA) of microbial community composition by sampling day and meat cut.**

PERMANOVA based on Bray–Curtis dissimilarities evaluating the contributions of sampling day and meat cut to microbial community composition. Sampling day and meat cut were evaluated as explanatory terms, with permutations restricted within butcher source. Models were fitted with terms entered in both sequential orders to assess order-dependent contributions. The table reports degrees of freedom, sums of squares, explained variance ($R^2$), pseudo-$F$ statistics, and permutation-based *p*-values.

**Table S3, Sheet 6. Pairwise PERMANOVA comparisons by sampling day and meat cut.**

Pairwise PERMANOVA comparisons of microbial community composition between day 0 and day 7 and among meat cut pairs, including back versus leg, back versus shoulder, and leg versus shoulder based on Bray–Curtis dissimilarities. The table reports degrees of freedom, sums of squares, explained variance ($R^2$), pseudo-$F$ statistics, permutation-based *p*-values, and false discovery rate-adjusted results.

**Table S3, Sheet 7. Comparison of within-pair and between-pair Bray–Curtis dissimilarities.**

Wilcoxon rank-sum comparison of within-pair Bray-Curtis dissimilarities (each day 0 sample compared with its own matched day 7 sample) against between-pair dissimilarities (each day 0 sample compared with day 7 samples from non-matched pairs). The table reports the Wilcoxon test statistic and *p*-value, along with the median Bray–Curtis dissimilarities for each comparison group.

**Table S4, Sheet 1. Phylum-level taxonomic composition and relative abundance across retained samples.**

Phylum-level taxonomic composition of the microbial communities from retained samples. The table reports the bacterial phyla detected and their corresponding relative abundances within each retained sample.

**Table S4, Sheet 2. Genus-level taxonomic composition and relative abundance across retained samples.**

Genus-level taxonomic composition of the microbial communities from retained samples. For each sample, the table reports the bacterial genera detected and their corresponding relative abundances, including sequences grouped under "*Other*" where applicable.

**Table S4, Sheet 3. Phylum-level differential abundance analysis using MaAsLin2 with butcher source modeled as a random effect.**

Phylum-level differential abundance analysis performed using MaAsLin2, with sampling day and meat cut modeled as fixed effects and butcher source included as a random effect. Day 0 serves as a baseline and Back as the reference category. The table reports the associated metadata variable and comparison level, model coefficient, standard error, nominal $p$-value, false discovery rate-adjusted $q$-value, total number of samples analyzed, number of samples in which each phylum was detected, and taxonomic assignment. Only associations with $q < 0.05$ are reported.

**Table S4, Sheet 4. Genus-level differential abundance analysis using MaAsLin2 with butcher source modeled as a random effect.**

Genus-level differential abundance analysis performed using MaAsLin2, with sampling day and meat cut modeled as fixed effects and butcher source as a random effect. Day 0 serves as a baseline and Back as the reference category. The table reports the associated metadata variable and comparison level, model coefficient, standard error, nominal $p$-value, false discovery rate-adjusted $q$-value, total number of samples analyzed, number of samples in which each genus was detected, and the corresponding taxonomic classification. Only associations with $q < 0.05$ are reported.

**Table S4, Sheet 5. ASV-level differential abundance analysis using MaAsLin2 with butcher source modeled as a random effect.**

ASV-level differential abundance analysis performed using MaAsLin2, with sampling day and meat cut modeled as fixed effects and butcher source included as a random effect. Day 0 serves as a baseline and Back as the reference category. For each associated ASV, the table reports the metadata variable and comparison level, model coefficient, standard error, nominal $p$-value, false discovery rate-adjusted $q$-value, total number of samples analyzed, number of samples in which the ASV was detected, and corresponding taxonomic classification. Only associations with $q < 0.05$ are reported.

**Table S5, Sheet 1. Breakaway estimates of species richness after application of a 3,000-read threshold.**

Sample-level species richness estimates generated using Breakaway for the 32 samples retained after applying the stricter minimum read-depth threshold of 3,000 reads. For each sample, the table reports the estimated richness, the associated estimation error, the lower and upper bounds, and the statistical model (Kemp, Negative Binomial, or Poisson) used for estimation.

**Table S5, Sheet 2. Betta random-effects model of Breakaway-estimated species richness after application of the 3,000-read threshold.**

Sensitivity analysis using a Betta random-effects model to evaluate Breakaway-estimated species richness among the 32 samples retained after the 3,000-read threshold. Sampling day and meat cut were modeled as fixed effects, with butcher source treated as a random effect. Day 0 and Back serve as the reference categories. The table reports coefficient estimates, standard errors, corresponding *p*-values, and model variance and covariance components.

**Table S5, Sheet 3. Homogeneity of multivariate dispersion after application of the 3,000-read threshold.**

Sensitivity analysis of multivariate dispersion among the 32 samples retained after the 3,000-read threshold. Bray–Curtis distances to group centroids were compared separately by sampling day, meat cut, and butcher source using 999 unrestricted permutations. The table reports degrees of freedom, sums of squares, mean squares, $F$ statistics, and permutation-based *p*-values.

**Table S5, Sheet 4. PERMANOVA of microbial community composition after application of the 3,000-read threshold.**

Sensitivity analysis of microbial community composition among the 32 samples retained after the 3,000-read threshold using PERMANOVA based on Bray–Curtis dissimilarities. Sampling day and meat cut were evaluated as explanatory terms, with permutations restricted by butcher source. The terms were entered in both sequential orders to assess order-dependent contributions. The table reports degrees of freedom, sums of squares, explained variance ($R^2$), pseudo-$F$ statistics, and permutation-based *p*-values based on 999 permutations.

**Table S5, Sheet 5. Betta fixed-effects model of Breakaway-estimated species richness by sampling day, meat cut, and butcher source.**

Betta sensitivity model in which sampling day, meat cut, and butcher source were all modeled as fixed effects. Day 0, Back, and Butcher 1 serve as the reference categories. The table reports coefficient estimates, standard errors, corresponding *p*-values, and model variance and covariance components.

**Table S5, Sheet 6. Bayesian fixed-effects model of Breakaway-estimated species richness by sampling day, meat cut, and butcher source.**

Posterior estimates from a Bayesian sensitivity model evaluating Breakaway-estimated species richness with sampling day, meat cut, and butcher source included as fixed effects. Uncertainty

in the sample-level richness estimates was incorporated using a Student-*t* likelihood. Day 0 serves as the baseline, while Back and Butcher 1 are the reference levels. The table reports posterior coefficient estimates, posterior standard errors, 95% Bayesian credible intervals (CrIs), residual distribution parameters, Rhat, and effective sample size diagnostics.

**Table S5, Sheet 7. Betta fixed-effects model of Shannon diversity by sampling day, meat cut, and butcher source.**

Betta sensitivity model in which sampling day, meat cut, and butcher source were all modeled as fixed effects. Day 0, Back, and Butcher 1 serve as the reference categories. The table reports coefficient estimates, standard errors, corresponding *p*-values, and model variance and covariance components.

**Table S5, Sheet 8. Bayesian fixed-effects model of Shannon diversity by sampling day, meat cut, and butcher source.**

Posterior estimates from a Bayesian sensitivity model evaluating Shannon diversity with sampling day, meat cut, and butcher source included as fixed effects. Uncertainty in the sample-level Shannon diversity estimates was incorporated using a Student-*t* likelihood. Day 0 serves as the baseline, and Back and Butcher 1 are the reference categories. The table reports posterior coefficient estimates, posterior standard errors, 95% Bayesian credible intervals (CrIs), residual distribution parameters, Rhat, and effective sample size diagnostics.

**Table S5, Sheet 9. Betta fixed-effects model of Simpson diversity by sampling day, meat cut, and butcher source.**

Betta sensitivity model in which sampling day, meat cut, and butcher source were all modeled as fixed effects. Day 0, Back, and Butcher 1 serve as the reference levels. The table reports coefficient estimates, standard errors, corresponding *p*-values, and model variance and covariance components.

**Table S5, Sheet 10. Bayesian fixed-effects model of Simpson diversity by sampling day, meat cut, and butcher source.**

Posterior estimates from a Bayesian sensitivity model evaluating Simpson diversity with sampling day, meat cut, and butcher source included as fixed effects. Uncertainty in the sample-level Simpson diversity estimates was incorporated using a Student-*t* likelihood. Day 0 serves as the baseline, and Back and Butcher 1 are the reference categories. The table reports posterior coefficient estimates, posterior standard errors, 95% Bayesian credible intervals (CrIs), residual distribution parameters, Rhat, and effective sample size diagnostics.

**Table S5, Sheet 11. Phylum-level differential abundance analysis with butcher source modeled as a random effect.**

Phylum-level differential abundance analysis performed using MaAsLin2, with sampling day and meat cut included as fixed effects and butcher source modeled as a random effect. Day 0 serves as a baseline and Back as a reference category. The table reports the phylum-level taxon, associated metadata variable, and comparison level, model coefficient, standard error, nominal

*p*-value, false discovery rate (FDR)-adjusted *q*-value, total number of samples analyzed, and the number of samples in which each phylum was detected. The table includes the complete model output.

**Table S5, Sheet 12. Phylum-level differential abundance analysis with butcher source modeled as a fixed effect.**

Phylum-level differential abundance analysis performed using MaAsLin2, with sampling day, meat cut, and butcher source included as fixed effects. Day 0 serves as a baseline, and Back and Butcher 1 as the reference categories. The table reports the phylum-level taxon, the associated metadata variable and comparison level, the model coefficient, standard error, nominal *p*-value, false discovery rate (FDR)-adjusted *q*-value, the total number of samples analyzed, and the number of samples in which each phylum was detected. The table includes the complete model output.

**Table S5, Sheet 13. Genus-level differential abundance analysis with butcher source modeled as a random effect.**

Genus-level differential abundance analysis performed using MaAsLin2, with sampling day and meat cut included as fixed effects and butcher source modeled as a random effect. Day 0 serves as a baseline and Back as a reference category. The table reports the genus-level taxon, the associated metadata variable and comparison level, the model coefficient, standard error, nominal *p*-value, false discovery rate (FDR)-adjusted *q*-value, the total number of samples analyzed, and the number of samples in which each genus was detected. The table includes the complete model output.

**Table S5, Sheet 14. Genus-level differential abundance analysis with butcher source modeled as a fixed effect.**

Genus-level differential abundance analysis performed using MaAsLin2, with sampling day, meat cut, and butcher source included as fixed effects. Day 0 serves as a baseline, and Back and Butcher 1 as the reference categories. The table reports the genus-level taxon, the associated metadata variable and comparison level, the model coefficient, standard error, nominal *p*-value, false discovery rate (FDR)-adjusted *q*-value, the total number of samples analyzed, and the number of samples in which each genus was detected. The table includes the complete model output.

**Table S5, Sheet 15. ASV-level differential abundance analysis with butcher source modeled as a random effect.**

ASV-level differential abundance analysis performed using MaAsLin2, with sampling day and meat cut included as fixed effects and butcher source modeled as a random effect. Day 0 serves as a baseline and Back as a reference category. The table reports the ASV identifier, associated metadata variable and comparison level, model coefficient, standard error, nominal *p*-value, false discovery rate (FDR)-adjusted *q*-value, total number of samples analyzed, number of samples in which each ASV was detected, and taxonomic assignment where available. The table includes the complete model output.

**Table S5, Sheet 16. ASV-level differential abundance analysis with butcher source modeled as a fixed effect.**

ASV-level differential abundance analysis performed using MaAsLin2, with sampling day, meat cut, and butcher source included as fixed effects. Day 0 serves as a baseline, and Back and Butcher 1 as the reference categories. The table reports the ASV identifier, associated metadata variable and comparison level, model coefficient, standard error, nominal *p*-value, false discovery rate (FDR)-adjusted *q*-value, total number of samples analyzed, number of samples in which each ASV was detected, and taxonomic assignment where available. The table includes the complete model output.
