# Supplementary material for: Microbiome Dynamics of Camel Meat During Cold Storage Revealed by 16S rRNA Gene Sequencing
Source: Biology (Basel). 2026 Aug 27;15(17):1462. doi: 10.3390/biology15171462 (PMC13564619; doi:10.3390/biology15171462)
Supplement: Supplementary file 1 [file biology-15-01462-s001.zip › File S1.pdf]

Supplementary Information for "Microbiome Dynamics of Camel Meat during Cold Storage Revealed by 16S rRNA Gene Sequencing"

Alpha Diversity-based Sensitivity Analyses

To assess the robustness of the alpha diversity results, sensitivity analyses were conducted in which the Butcher source, treated as a fixed rather than a random effect, was included for all three indices using both Bayesian hierarchical modeling (Bayesian) and the betta() framework (betta). Here, I am presenting sensitivity and primary analysis results for Breakaway-estimated richness (Bayesian: β = −170.17, *95% CrI* [−227.37, −117.68]; betta: β = −177.77, p < 0.001; primary Bayesian: β = −162.20, *95% CrI* [−216.55, −110.80]) in which the day 7 effect remained significant and directionally consistent with the primary findings. Moreover, Shannon diversity showed the same pattern (Bayesian: β = −0.65, *95% CrI* [−1.01, −0.29]; betta: β = −0.65, p < 0.001; primary: β = −0.62, *95% CrI* [−1.00, −0.25]). Similarly, the Simpson index results (Bayesian: β = 0.02, *95% CrI* [0.01, 0.03]; betta: β = 0.024, p < 0.001; primary: β = 0.02, *95% CrI* [0.01, 0.03]) are also consistent with the primary analysis. Detailed results for primary and sensitivity analyses are reported in supplementary materials (Figure S4, Sheet 1, 2, and 3, Table S2 and Table S5).

Sensitivity Analysis for Differentially Abundant Features

Microbiome Multivariable Associations with Linear Models 2 (MaAsLin2) sensitivity analyses, in which butcher source was treated as a fixed effect, were performed at the phylum, genus, and ASV levels. At the phylum level, *Pseudomonadota* remained enriched at day 7 (*coefficient* = 0.974, q = 0.0378), whereas *Actinomycetota* (*coefficient* = −5.238, q = 1.73×10^{-9}) and *Bacteroidota* (*coefficient* = −3.341, q = 1.33×10^{-8}) were depleted, matching the primary random-effect model in direction and significance (Table S5, Sheet 12). At the genus level, the same 16 genera were significant under both models, with Butcher as a fixed and a random effect. *Pseudomonas* was the only genus enriched in the sensitivity analysis (coefficient = 2.6567, q = 0.0034), consistent with the primary analysis regarding storage duration. At the ASV level, all 59 ASVs significant in the primary model remained significant under the fixed-effect model. These results are consistent with the primary analysis (Table S5, Sheet 12, 14, and 15).

Sensitivity Analysis Based on 3000-read Threshold

To test whether the 1000-read inclusion threshold influenced the storage effect, alpha and beta diversities were reanalyzed using a stringent 3000-read cutoff. Of the original 54 samples, 32 passed this threshold. Richness was re-estimated with breakaway and tested using Bayesian hierarchical modeling, with day and cut as fixed effects and Butcher as a random effect (β = -166.25, 95% CrI [-237.70, -96.23]) (Table S5, Sheet 1 and 2). This finding was consistent with the results at the 1000-read threshold, as the day 7 effect remained significant relative to day 0. Homogeneity of multivariate dispersions also showed consistent results, with Butcher dispersion (F = 15.552, p = 0.001) the only significant term (Table S5, Sheet 3). Community structure was reassessed using PERMANOVA on Bray-Curtis dissimilarities with the same parameters, including day and cut as terms, and permutations restricted within Butcher; the day effect (R^2 = 0.09836, F = 3.2591, p = 0.012) remained significant, consistent with the 1000-read threshold findings (Table S5, Sheet 4).

Phylogenetic Context of Detected Taxa

Phylogenetic trees summarizing the evolutionary relationships among detected bacterial phyla and genera are provided for general taxonomic visualization. These trees illustrate the taxonomic breadth of the community characterized in this study but are not directly linked to the storage-duration comparisons. Hence, they are included as descriptive background rather than as interpretive or inferential evidence (Figure S4, Sheet 4 and 5).
